# Graphene-based hybrid composites for cancer diagnostic and therapy

**DOI:** 10.1186/s12967-024-05438-7

**Published:** 2024-07-02

**Authors:** Mahnaz Asadi, Seyed Hosein Ghorbani, Leila Mahdavian, Mohammad Aghamohammadi

**Affiliations:** 1 Department of Chemistry, Borujerd Branch, Islamic Azad University, Borujerd, Iran; 2 Department of Chemistry, Doroud Branch, Islamic Azad University, Doroud, Iran

**Keywords:** Graphene, Cancer therapy, Cancer diagnosis, Drug delivery, Graphene simulative responsive systems, Smart delivery

## Abstract

The application of graphene-based nanocomposites for therapeutic and diagnostic reasons has advanced considerably in recent years due to advancements in the synthesis and design of graphene-based nanocomposites, giving rise to a new field of nano-cancer diagnosis and treatment. Nano-graphene is being utilized more often in the field of cancer therapy, where it is employed in conjunction with diagnostics and treatment to address the complex clinical obstacles and problems associated with this life-threatening illness. When compared to other nanomaterials, graphene derivatives stand out due to their remarkable structural, mechanical, electrical, optical, and thermal capabilities. The high specific surface area of these materials makes them useful as carriers in controlled release systems that respond to external stimuli; these compounds include drugs and biomolecules like nucleic acid sequences (DNA and RNA). Furthermore, the presence of distinctive sheet-like nanostructures and the capacity for photothermal conversion have rendered graphene-based nanocomposites highly favorable for optical therapeutic applications, including photothermal treatment (PTT), photodynamic therapy (PDT), and theranostics. This review highlights the current state and benefits of using graphene-based nanocomposites in cancer diagnosis and therapy and discusses the obstacles and prospects of their future development. Then we focus on graphene-based nanocomposites applications in cancer treatment, including smart drug delivery systems, PTT, and PDT. Lastly, the biocompatibility of graphene-based nanocomposites is also discussed to provide a unique overview of the topic.

## Introduction

In recent years, nanotechnology and advanced nano-delivery systems have shown that can notably address some shortcomings of conventional therapeutics and the low efficacy treatments [1]. Poor solubility, low bioavailability, unexpected metabolization and drug elimination of the body before function on their desired sites, non-specific selectivity, and side effects are some of the drawbacks of traditional treatments [2, 3]. However, recent advances in nanotechnology and nanomaterials could have provided a better bio-distribution of drugs, reduced adverse effects, and delivered therapeutic agents to the targeted sites, improving bioavailability [4, 5]. In recent years, numerous organic and inorganic nanomaterials have been investigated for nanomedicine applications [6–9]. In this context, two-dimensional (2D)-carbon-based nanomaterials provide new prospects attracting the attention of many scientists due to their fascinating characteristics such as high surface area, easy functionalization, and acceptable biocompatibility [10, 11]. 2D-carbon-based materials have been differentiated based on the number of layers in their structure, oxygen-containing functional groups, and chemical composition [12]. Graphene, graphene oxide (GO), and reduced graphene oxide (rGO) are the most well-known and most frequently explored forms of 2D-carbon-based materials in the realm of cancer diagnosis and treatment [13].

Graphene is composed of a uniform monolayer of carbon atoms. Carbon atoms with hexagonal arrangements are bonded tightly in a honeycomb structure. In this structure, any carbon atoms are attached to two others with *sp*^*2*^ hybridization, and one out-of-plane p orbital supplies the electron network [14]. Graphene results of graphite exfoliation via chemical vapor deposition. As graphene does not contain any oxygen groups, it is considered hydrophobic. However, this unique structure leads to graphene’s interesting properties, including mechanical strength, and high thermal, electrical, optical, and magnetic features, making it a good candidate for different applications as well as cancer diagnosis and therapy [15]. To date, numerous 2D-graphene-based materials have been investigated; these materials range in size, chemical make-up, biodegradability, and compatibility with living systems [16]. 2D-graphene-based materials having such a wide range of properties hold great promise for use in biomedicine, notably in areas like biosensors, multimodal imaging, drug/gene delivery, and cancer treatment [17].

The 2D-graphene-based materials have a huge specific surface area, which makes them stand out. This property allows them to adsorb molecules efficiently via covalent or non-covalent interactions. As a result, they show more restraint in their discharge in response to environmental cues. 2D-graphene-based materials are of special interest for optical treatments including photothermal therapy (PTT) and photodynamic therapy (PDT) due to their sheet-like nanostructures. Additionally, these nanomaterials may respond to near-infrared (NIR) light. The advantages above have resulted in 2D-graphene-based materials becoming the prevailing nanoplatforms for theranostic investigations. In recent years, the primary focus of researchers has been on the fabrication or optimization of monotherapy. However, numerous preclinical and clinical studies have demonstrated that monotherapy does not yield the expected level of effectiveness. This is primarily attributed to the recurrence and metastasis of cancer. The combination of 2D-graphene-based nanocomposites with other functional moieties has the potential to create a novel category of therapeutic agents in the field of biomedicine. This integration can significantly enhance the properties of 2D-graphene-based nanocomposites, enabling synergistic applications in cancer diagnosis and therapy on the 2D-graphene-based nanomaterials [18].

This stydy provides an overview of the present status and advantages associated with the utilization of 2D-graphene-based nanomaterials in the field of cancer detection and therapy. Additionally, it explores the challenges encountered and the potential for future advancements in this area. In this discussion, we will examine three highly promising applications of 2D-graphene-based nanomaterials in the field of cancer treatment. These applications include: (1) cancers diagnosis, (2) drug delivery, (3) PTT, and (4) PDT. This study also presents a summary of the biocompatibility of 2D-graphene-based nanomaterials, offering valuable insights into this subject matter.

## Graphene-based hybrid composites for cancer diagnosis

Nowadays, cancer is one of the human health threats in the world due to its high prevalence and death [19]. It is predicted that the early diagnosis of cancer can attenuate its death rate. Magnetic resonance imaging (MRI) and Computed Tomography (CT) scan are the most conventional methods used for cancer detection [20]. Although these techniques have been to a degree of success, the lack of effective techniques with acceptable sensitivity and inefficiency for early detection of cancer still remains a major concern. Recent studies have stated that 2D-graphene-based materials combined with many contrast agents can provide a new platform to detect tumor tissues to achieve the necessity of multimodal imaging [21]. Bio-imaging and biosensors possess vital functions helping to perceive different biological cellular and subcellular processes. 2D-graphene-based biosensors can assist the existing techniques to become more powerful improving cancer diagnosis at the early stage and monitoring the efficacy of treatments [20]. Herein, it was focused on 2D-graphene-based materials used in different biosensing techniques and bioimaging methods.

### Biosensing

Biosensors are analytical tools constructed of two parts including a receptor (biological unit) and a transducer (electronic unit) which enable to detect the small molecules and large biomolecules [22, 23]. They can qualitatively or quantitatively recognize specific types of analytes using various techniques such as spectrochemical, electrochemical, magneto-chemical, fluorescence resonance energy transfer (FRET), surface-enhanced Raman scattering (SERS), fluorescence spectroscopy, and surface plasmon resonance (SPR). They are mostly used for detecting chemical analytes and biomolecules that have a key role in the disease process, so their detection is very crucial in diagnosis and therapy [22]. Graphene-based materials have good electrochemical and optical properties for biosensing detection applications. they can interact with various molecules via p–p stacking or electrostatic interaction [24]. Additionally, their surface can be modified to allow them to interact with defined analytes. GO is more explored for biosensing among graphene derivatives due to its acceptable biocompatibility, good water dispersibility, surface functional groups, and its G-band in Raman spectra [25]. GO-based biosensors can detect lower limits, respond rapidly, have high sensitivity, and improve signal-to-noise ratios [21, 24].

#### Electrochemical biosensor

The electrochemical sensor has been presented extensive opportunities for detecting several analysts such as cancer biomarkers. Their most notable characteristics include high selectivity, sensitivity, accuracy, and easy procedure [26]. On the other hand, tumor cells usually have different specific biomarkers that can be detected by biosensors helping to early detection of cancer. These biomarkers are included proteins, hormones, enzymatic, embryonic antigens, carbohydrate antigens, isoenzymes, oncogenes, etc. In this context, various types of electrochemical biosensors have been established using graphene-based materials. For example, a new disposable electrochemical biosensor was fabricated for detecting a specific DNA sequence on the p53 tumor suppressor gene (TP53) [27]. The electrochemical part consisted of screen-printed carbon electrodes (SPCEs) functionalized with rGO–carboxymethylcellulose (rGO-CMC). Single nucleotide polymorphism in cDNAs from human breast cancer cell lines was discriminated by this platform, which can be an excellent diagnostic device in clinical analysis. Furthermore, an electrochemical DNA probe immobilizedMoS2/graphene-based sensor was developed for detecting circulating tumor DNA. In comparison to other methods for the detection of circulating tumor DNA, this biosensor showed high sensitivity not needing labeling and using amplifiers [28].

As miRNAs have a key role in the early diagnosis of cancer, several graphene-based electrode materials with different structures and composites have been developed for the detection of various types of microRNAs. For instance, a disposable pencil graphite electrode modified with graphene was developed for mir-21 detection [29]. This study reported that the graphene-modified electrode had a 2.77 times lower detection limit (3.12 pmol) compared with the unmodified electrode. In addition, this graphene-modified electrode did not require for sample preparation and amplification process before the detection of real samples. Another electrochemical biosensor was fabricated for plasma miR-155 detection by M.Azimzadeh et al. [30]. They fabricated a thiolated glassy carbon electrode whose surface was modified with GO sheets decorated with gold nanorods (GNRs). They reported that for the range of 2.0 fM to 8.0 pM, the biosensor signal relationship with miRNA concentration was linear and the detection limit was 0.6 fM. In addition, this system showed high selectivity, good sensitivity, storage ability, and acceptable response, and did not need any purification and amplification process before the detection of real samples. A label-free electrochemical immune-sensor functionalized with polymer-rGO was fabricated for the detection of miRNA-29b-1 and miRNA-141 [31]. This immune-sensor displayed excellent stability, good sensitivity with a limit of quantification of 8 fM, and high selectivity as it discriminates mismatch.

Proteins are other types of biomarkers that can be measured in tissues and blood serum for early diagnosis of cancer. Any changes in protein levels indicate an alteration in their structure or post-translation process related to the existence and development of cancer cells. So, the detection of protein biomarkers is vital for diagnosis, drug screening, and treatment approach evaluation [32]. In this context, various graphene-based biosensors were established to detect a protein biomarker related to cancer cells [33]. For example, a biosensing platform was generated of porphyrinnon- functionalized graphene-modified glassy carbon electrode for clinical diagnosis of cyclin A2 which is a prognostic indicator in early-stage cancers [34]. Using graphene in this platform improves the sensor function due to its instinct conductivity and providing oxygen-containing functional groups for the following immobility of peptide. A chitosan-modified-rGO-based biosensor was fabricated for the VEGFR2 detection with acceptable susceptibility and a detection limit of 0.28 pM [35]. It could directly measure the total amount of VEGFR2 in cell lysates and also monitor VEGFR2 expression changes stimulated by different inhibitor treatments.

Reactive oxygen species (ROS) are essential small biomolecules for various physiological processes and signaling pathways. In abnormal conditions such as cancer, ROS level is changed caused by oxidative stress [36]. One of the reactive oxygen species is H_2_O_2_ molecules that are directly related to protein production, apoptosis, and DNA damage [189]. In this concept, a glassy carbon electrode modifying with a graphene-based nanocomposite (rGO-Au-poly(toluidine blue O) films) was fabricated to detect H_2_O_2_ [37]. This platform was sensitive to H_2_O_2_ with a detection limit of 0.2 µM considered a potential biosensor for various cancer cell detection. Pt–MnO_2_-rGO-based biosensor is another example that was fabricated for the detection of H_2_O_2_ molecules from cells with high stability and sensitivity with a detection limit of 1.0 µM [38]. Regarding these studies, it is suggested that the incorporation of graphene-based materials in the electrochemical biosensor can provide new platform for detecting various cancer biomarkers with highly sensitive, accuracy, stability, and reproducibility.

#### Fluorescence sensor

In several disease conditions including cancer, the cellular and subcellular metabolites, ions, and molecules are changed. Thus, screening these molecules afford critical information to appreciate many conditions. Fluorescent technology can provide an effective platform for real-time monitoring, measuring, and detecting these molecules [39]. In this regard, various fluorescent biosensors incorporated with graphene-based materials have been fabricated for cancer detection. In this regard, various fluorescent biosensors incorporated with graphene-based materials have been fabricated for cancer detection. A fluorescent aptasensor was developed to detect Mucin1, an epithelial tumor marker [40]. To this aim, The GO surface was modified with the MUC1 (target analyte) and Cy5-modified aptamer. In the absence of MUC1, the fluorescence of a single-stranded dye-labeled MUC1 was quenched by GO while upon adding MUC1 the fluorescence is recovered, and MUC1 could be identified. In another study, a fluorescent aptasensor based on GO was designed for detection of leukemia. The surface of GO was modified with carboxy-fluorescein-labeled Sgc8 aptamer. The fluorescence of the system was quenched through FRET. The fluorescence was quenched when target molecules was absent in medium. The quenched fluorescence was recovered upon to present of the target molecules by adding the CCRF-CEM cells. Thus, the number of CCRF-CEM cells detected based on the fluorescence signals intensity with a detection limit of 10 cells/mL [41]. Regarding, GO can be considered a great energy receptor in FRET-based biosensors widely applicable in fluorescence aptasensor [42]. In addition, using graphene-based material in aptamers can improve their stability compared to the free aptamer probe [43].

#### Field-effect transistor (FET) biosensor

The FET biosensors are efficient and promising biomedical tools. In these technologies, fluorescent dyes or electrochemical tags are not necessary. They are relatively fast, have label-free detection, and act sensitively and selectively because of the interfacial transfer of charge [20]. However, their effective electrical function, their structure, and the design of an active channel layer are still challenges. In this context, graphene-based materials are explored for FRET biosensors due to their electronic properties. For example, an rGO-based FET biosensor was fabricated for peptide nucleic acid (PNA)–DNA detection [44]. To this aim, the surface of the sensor was modified with the prepared rGO suspension by drop-casting approach. PNA was applied as the capture probe, and DNA was detected by PNA–DNA hybridization on the rGO-based FET biosensor. This biosensor showed high sensitivity with a detection limit of 100 fM, high specificity, and reproducibility in cancer diagnosis. Another study was performed to develop a gold nanoparticles (AuNPs)-rGO-based FET biosensor for miRNA detection [45]. The sensor surface was cast by rGO suspension and then decorated with AuNPs. Next, the PNA probe was immobilized on the AuNPs-rGO surface. The miRNA was determined by the PNA-miRNA hybridization with high sensitivity.

### Bioimaging

Bioimaging is an important aspect of cancer diagnosis, as it can monitor the biological conditions in both in vivo and in vitro. In principle, materials applying bioimaging should be non-toxic, biocompatible, sensitive, and have high specificity [26]. To this, graphene-based materials possess several advantages that attract much attention to exploring bioimaging applications including capability in real-time imaging, non-ionizing energy, economical availability, in vivo photothermal effect in both NIR windows (650–950 nm, and 1000–1350). Although these materials have shown acceptable toxicity for bioimaging applications, however, their selectivity and sensitivity should be addressed.

Graphene-based materials have been used as fluorescence probes to detect various diseases, mainly cancer cells. For example, rGO modified with quantum dots and folic acid (FA) was assessed for fluorescent bioimaging in cancer cells [46]. It was reported that rGO can convert NIR radiation into heat causing simultaneously cell death (photothermal effect) and fluorescence quenching. Thus, this system was used for PTT, tumor imaging, and in situ monitoring of treatment. Additionally, antibody-conjugated GO was applied as a Raman probe for the selective identification of breast cancer cells (SKBR3) [47]. The results demonstrated fabricated GO-based probe can discriminate SKBR3 cells in tumor cell mixtures with the detection limit of 60 cell/mL. The fluorescence signal of GO, rGO, and partially reduced graphene oxide (prGO) was investigated in liver cancer cells by means of DUV fluorescence bioimaging [48]. It was reported that the prGO remarkably increased the cell fluorescence and enhanced the signal intensity (2.5 times). This result suggests that the prGO nanosheet can be used simultaneously for loading therapeutic agents and monitoring drug delivery by fluorescence microscopy. Beside 2D- graphene-based materials, graphene quantum dot is a derivative of graphene-based materials with a zero-dimension structure that is most widely used for fluorescence bioimaging [49]. GQDs hold great promise for bioimaging applications due to their strong and tunable photoluminescence, photostability, and biocompatibility.

## Graphene-based hybrid composites for cancer drug delivery

The main purpose of fabricating a drug delivery system (DDS) for cancer treatment is to deliver anti-cancer agents inside tumor tissue to improve efficiency with fewer side effects on normal cells. The low water solubility of chemotherapeutics and their non-specificity localization in the tumor site make side effects on normal tissues [14]. In recent years, nanotechnology progress has facilitated these difficulties by introducing various nanocarriers with efficient functions in DDS [50]. Owing to the high surface area, acceptable biocompatibility, and facility of their surface modification, graphene-based materials have been widely investigated as imaging contrast agents and drug/gene delivery systems, and bacterial inhibitors. Additionally, their ability in the photothermal conversion of the NIR wavelengths provides a platform for PTT [51].

Graphene-based materials have been shown promising potential for nanocarrier usage due to their uniform small size, high surface area, low cost, intrinsic optical characteristics, and their ability to establish non-covalent interactions with aromatic components [52]. π-π stacking, hydrogen bonding, and hydrophobic or electrostatic interactions are non-covalent interactions of these materials that can support high loading of less soluble chemotherapeutics with effective efficiency. In recent years, several investigations have been directed at the delivery of hydrophobic chemotherapeutics via simple physisorption through π-π stacking. Doxorubicin (DOX) and paclitaxel (RTX) are hydrophobic chemotherapeutics that are successfully loaded on GO via π-π interactions to enhance cytotoxicity and inhibit cancer cells [53, 54].

In addition, the covalent modification of rGo and GO sheets is possible due to the existence of some defects in their lattice and oxygen-containing groups on their surface. These modifications can be accessed via different approaches such as condensation/addition and nucleophilic/ electrophilic reactions [55]. These approaches can be useful to achieve nanocarriers with decent biocompatibility and controllable behavior in biological mediums. For instance, polyethylene glycol (PEG) is the most common polymer covalently functionalized on the GO sheets to improve in vivo pharmacokinetics, enhance biocompatibility, and alleviate non-specific interactions with biological medium, molecules, and cells [56, 57]. Poly(vinyl alcohol) (PVA) [58], polysebacic anhydride (PSA) [59], poly(N-isopropylacrylamide) (PNIPAM) [60], polyethylenimine (PEI) [61, 62], chitosan [63, 64] and amphiphilic copolymers [65] are other polymers that have been covalently functionalized on GO sheets to enhance biocompatibility.

In addition to the improvement of biocompatibility, surface modification can be used for conjugate graphene-based materials with special ligands providing a platform for targeted DDS recognizable by tumor cells. Targeted delivery is a well-known approach to enhance the concentration of anti-cancer agents in the tumor side leading to improving their therapeutic efficacy and decreasing their side effects. In this context, several targeting ligands namely peptides [66], FA [67], aptamers [68], and monoclonal antibodies [69] have been investigated using graphene-based materials as nanocarriers for cancer therapy. Furthermore, functionalized graphene-based materials are talented candidates for designing DDS with multifunctional applications. In this respect, the most recent progress in DDS usage of graphene-based materials specifically for smart delivery, PTT, and PDT applications is discussed in the following sections.

**Table 1:** Various applications of G-based hybrid composites in cancer therapy

Application	Type of Graphene-based nanomaterial	Anti-cancer agent	Size (nm)	Release mode	Achievements	Refs.
Targeted DDS	NGO-PEG-HN-1	DOX	122	Thermal-sensitive drug release	-Increasing cellular uptakes and cytotoxicity in OSCC cells compared to free drug.	[70]
	GO-PEI-PEG-CPP	Rictor siRNA	195–230	-	-Increasing the cellular internalization -Improved inhibition effect of siRNA on the cancer cell viability and increase in vitro apoptosis -Targeting ability significantly repressed the tumor growth in comparison with the non-targeted one or free siRNA	[71]
	Fe_3_O_4_–graphene	5-fluorouracil	5–13	pH-responsive drug release	-pH dependent drug release -Enhancement of cell internalization with remarkable biocompatibility	[72]
	GO-Chitosan-FA	Camptothecin / 3,3′Diindolylmethane	230–355	Controlled drug release	-Co-delivery of two anti-cancer drugs making a synergistic effect in breast cancer treatment	[73]
	GO-PEG-FA	Camptothecin	100–200	Controlled drug release	-pH-dependent drug release -Enhancement of the anticancer activity	[74]
pH-sensitive /targeted DDS	GO-Tf	Dihydroartemisinin	100–200	pH-sensitive drug release	-Enhanced tumor delivery specificity -Increasing cytotoxicity -Improvment in vivo efficiency with minimal side effects	[75]
pH-sensitive DDS	GO–PVP–Fe_3_O_4_	Quercetin	∼100 μm	pH-responsive drug release	-pH-responsive controlled drug release -Enhancement of the cytotoxicity than the free drug	[76]
	Pectin-GO-Fe_3_O_4_	Paclitaxel	6–12	pH-sensitive drug release	-Improved drug loading capacity - pH-responsive drug release	[77]
	GO-CMC	DOX	-	Sustained drug release	-acceptable biocompatibility -pH-responsive controlled drug release	[78]
	CMC-starch-rGO	Curcumin	-	-	-pH-responsive sustained drug release	[79]
Redox-Stimuli DDS	NGO-SS-mPEG	DOX	200	pH responsive drug release	-Increasing the DOX release percentage upon adding GSH Inducing more growth inhibitory effect on cancer cells	[80]
	Ag@GO-PEI-SS-DOX	DOX	100–400	Stimulate responsive system	-GSH-stimulated drug release -Ability to monitoring cellular uptake via SERS technique	[81]
	rGO/QC-PEG/Plu-SH	DOX	140–293	Controlled drug release	-Triggered pH/redox responsive drug release	[82]
	GO-SS-Ce6	Chlorin e6 Photosensitizer	103	Redox drug release	Increasing lethal effect upon exposure with laser irradiation compared to free Ce6 in the same condition	[83]
Thermo- Stimuli DDS	PNIPAM-GS	Camptothecin	189	Sustained drug release	-loading water-insoluble anticancer agents with thermos- dependent release profile - In vitro high lethal effect on cancer cells	[60]
	SMGO/P(NIPAM-co-AA) NGs	DOX	83	-	-Thermo/pH dependent releasing behavio -Acceptable biocompatibility -Enhanced cytotoxicity compared to free DOX	[84]
	Chitosan-GO	DOX	35	Photothermal drug release	-Thermo-triggered DOX release - Significantly greater cytotoxicity in cancer cells	[85]
Magnetic DDS	γ-Fe_2_O_3_-NGO	Cisplatin	350–400	Sustained drug release	-High loading capacity with sustained release - Magnetic actuation for targeting delivery	[86]
	GO-HA- Fe_3_O_4_	DOX/ paclitaxel	130–160	-	-Targeted Drug Delivery - Combination therapy -Synergic anti-cancer effect via Magnetothermal Therapy and chemotherapy	[87]
	GO-IONP-PEG-	DOX	50–300	-	-Magnetically targeted delivery - localized PTT -MR imaging	[88]
	PVP/PVA- GO-Fe_3_O_4_	DOX/ Paclitaxel	14–500	Triggered drug release	-Combination therapy -Synergic anti-cancer effect via thermo-Chemotherapy	[89]
	GO-Gd- mAb	DOX	40–180	sustained and pH-sensitive release	-Targeted Drug Delivery -MR imaging	[90]
	Chitosan- Fe_3_O_4−_graphene	DOX	126	pH-dependent drug release	-Co-delivery of drugs and gene -MRI contrast agents -Combination of chemo- and gene therapy	[91]
	β-CD-GO-Fe_3_O_4_	DOX/ Epirubicin	13	Controlled drug release	-Magnetically targeted delivery -pH-controlled release	[92]
	CS/SA-GO-Fe_3_O_4_	DOX	40–60	pH-sensitive drug release	-pH-controlled release -Magnetically targeted delivery -Combination of chemotherapy and PTT	[93]
	GO/SPION/PLGA	5-iodo-2-deoxyuridine (IUdR)	72	-	-Magnetic targeting delivery -Improving efficiency of IUdR as a radiosensitizer -MR imaging	[94]
	Fe_3_O_4_-rGO	DOX	8–10	Sustained drug release	-Magnetically targeted delivery -Combination of chemo-thermo therapy	[95]
PTT	UCNPs-GO	ZnPc	28–40	-	- Upconversion luminescence imaging - Combinatorial PDT/PTT of cancer	[96]
	GO-poloxamer 188	DOX/ Irinotecan	212	pH-sensitive drug release	- Dual-Drug Chemo-PTT - Higher therapeutic efficacy to overcome resistance to chemotherapeutics.	[97]
	GO@Gd-PEG-FA	DOX	-	-	-MR imaging -tumor targeting - Photothermal-Chemotherapy	[98]
	rGO/PEG	Resveratrol	100–400	Stimulate responsive system	-A synergistic action of PTT in combined with the Resveratrol effect	[81]
	rGO-AuNRVe)	DOX	65	Controlled drug release	-pH/NIR triggered release -An intensive lethal effect resulted of Photothermal-Chemotherapy	[99]
PDT	Magnetic rGO- allylamine-g- 4-hydroxycoumarin	Camptothecin	-	pH-responsive drug release	-pH-sensitive drug-release -Higher toxicity effect against MCF-7 cell line compared with the normal fibroblast cell line (WS-1)	[100]
	PEG–GO–FA/ICG	Ginsenoside Rg3	-	-	-Increased therapeutic efficacy -Decreased tumor development after NIR light irradiation	[101]
	GO-PEG-OSA	PTX	-	pH-sensitive drug release	– The inhibition of the p-glycoprotein pump leads to the suppression of resistance to PTX by inducing the generation of ROS by NIR irradiation – The deactivation of the PGP pump occurred due to decreased ATP synthesis resulting from elevated levels of ROS and mitochondrial damage -Increased cell death	[70]

NGO: nano GO; HN-1: TSPLNIHNGQKL; CPP: cell-penetrating peptide; Tf: Transferrin; SERS: Surface-Enhanced Raman Scattering; QC: 2-chloro-3´,4´-dihydroxyacetophenone quaternized poly (ethylene glycol)-g-poly(dimethylaminoethyl methacrylate); Plu-SH: Pluronic; Ce6: Chlorin e6 Photosensitizer; PNIPAM: poly(*N*-isopropylacrylamide); GS: graphene sheets; SMGO: salep modified GO; AA: acrylic acid; NGs: Nanogels; PVP: polyvinylpyrrolidone; HA: Hyaluronic Acid; mAb: monoclonal antibody; β-CD: β-cyclodextrin; CS/SA: chitosan/sodium alginate; PLGA: poly (lactic-co-glycolic acid); ZnPc: phthalocyanine; UCNPs: upconversion nanoparticles; AuNRVe: gold nanorods shell-coated vesicle; OSA: oxidized sodium alginate

### Smart drug delivery

Another strategy in the modification of graphene-based materials is approaching to achieve a smart DDS being able to recognize a type of environmental stimuli and respond to it. It has been well-known that tumor environments have some differences in physicochemical properties of microenvironments such as temperature, pH, expressing several receptors, and enzymes compared to normal cells [102]. These differences are considered to develop the smart DDS that can release their cargo upon reaching the targeted site. To this aim, graphene-based materials have gained popularity in smart DDS that are reviewed in the following based on the types of their stimulus.

#### pH-sensitive graphene-based DDS

One of the most common strategies for fabricating smart DDS is pH modulation in cancer targeting. An abnormal property of tumor tissues is protons accumulation in the extracellular matrix leading to an acidic environment around the tumor regions that are not present in normal cells. This difference has been widely explored for pH-sensitive DDS in cancer treatments. In pH-sensitive graphene-based carriers, the anticancer drugs are commonly loaded on their platforms via simple physisorption (such as π-π stacking, hydrogen bindings, and hydrophobic interactions). These interactions between loaded drugs and graphene surfaces are weakened upon exposure to the acidic environment resulting in the loaded drug becoming released at lower pH [103]. One of the first pH-stimuli graphene-based DDS was PEG functionalized GO (PEG-GO) for DOX delivery in cancer therapy [104]. It was reported that PEG-GO released about 40% of DOX in an acidic solution (pH = 5.5) after 24 h while it released only 15% of DOX in a physiological pH = 7.4 over 48 h. Moreover, an anti-CD20 antibody (Rituxan) was also conjugated to the PEG-GO for B-cell-specific bonding. In vitro Cytotoxicity results showed Rituxan conjugating PEG-GO/DOX significantly increased cell growth inhibition rate in compared to non-Rituxan PEG-GO/DOX nanocarrier. In another example, poly (2-(diethylamino) ethyl methacrylate) (PDEMAEMA) was conjugated to GO to achieve a pH-sensitive nanocarrier used for loading camptothecin (CPT) [103]. This pH-triggered DDS was examined in three buffer solutions with different pH (pH = 5.5, 7.4, and 9.0). The maximum drug release occurred at pH = 5.5 over 48 h (~%60) while the drug release was insignificant at higher pH. In addition, the cytotoxicity assay on N2a cells revealed that the PDEMAEMA-GO did not show a toxicity effect while CPT-loaded-PDEMAEMA-GO had a ~%70 cell growth inhibition.

Arg-Gly-Asp peptide (RGD) and hyaluronic acid (HA) were grafted GO (GO-HA-RGD) to fabricate a PH-sensitive dual-receptor targeting DOX delivery [105]. In vitro, study revealed that this system had a pH-sensitive and sustained release. The competitive assay showed that Dox@ GO-HA-RGD had stronger cytotoxicity than Dox@GO-HA and DOX@GO in the SKOV-3 cell line. However, the GO-HA-RGD demonstrated excellent biocompatibility in the HOSEpiC and SKOV-3 cell lines. Furthermore, it was reported that HA and RGD conjugation could effectively increase the cellular uptake of the GO-HA-RGD in the SKOV-3 cell line through a synergic effect of CD44-HA and integrin-RGD facilitated endocytosis. In addition to the over mentioned examples, chitosan [106, 107], pluronic F127 [65], carboxymethyl callous (CMC) [78], and poly(vinylpyrrolidone) (PVP) [76]are other examples of polymers that are grafted to the GO surface aiming to pH-sensitive DDS fabrication (Table 1). In most of these studies, it has been shown that DDS based on GO can be modified to be a multifunctional platform simultaneously possessing several capabilities such as targeting and imaging in addition to being pH-sensitive.

#### Redox-stimuli graphene-based DDS

**Fig. 1:**
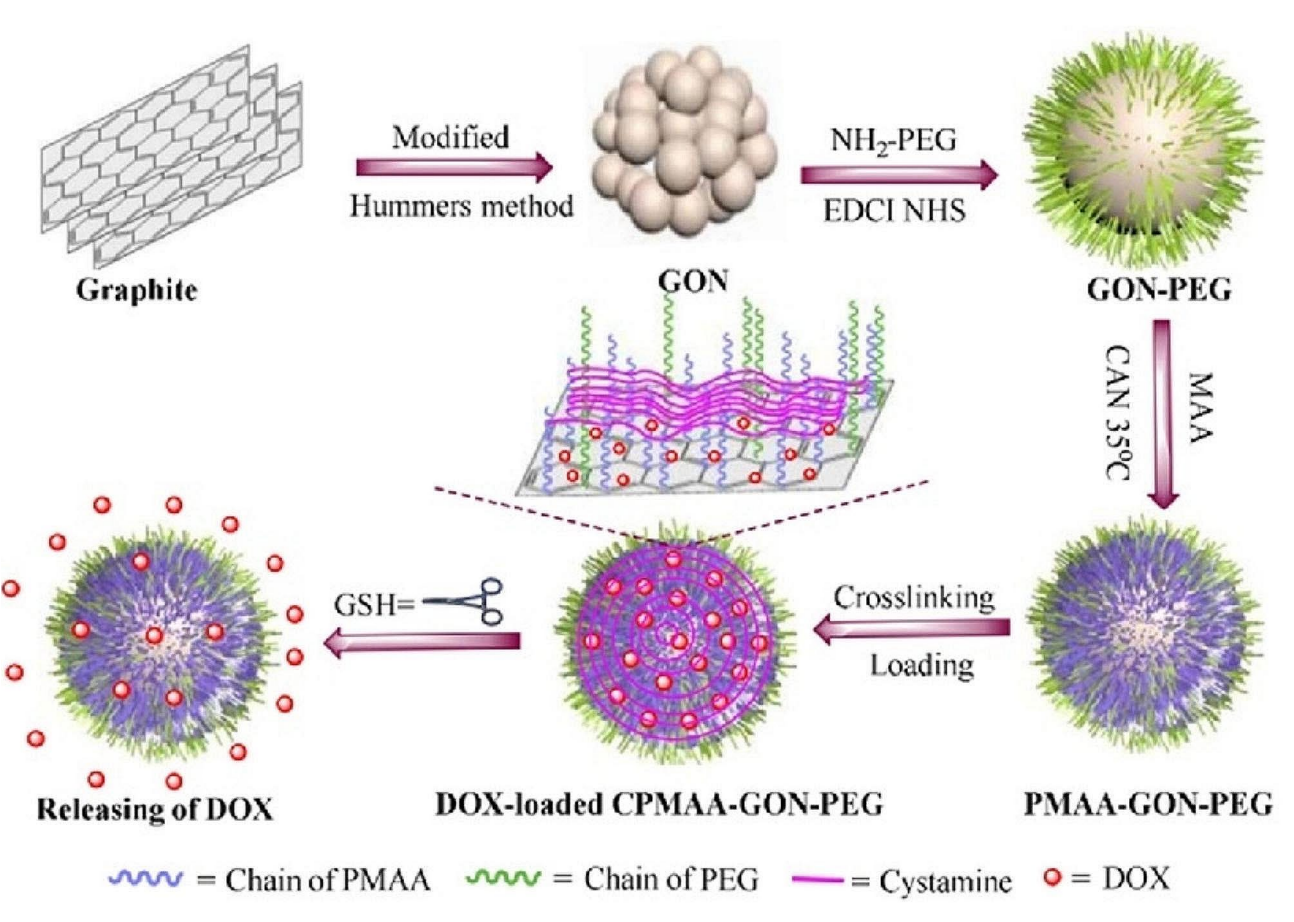
Schematic illustration of the preparation, DOX loading and redox-stimuli release of the CPMAA-GON-PEG. DOX release in acidic and high GSH level conditions [109]. Note: Copyright ©2015. ACS

Glutathione (GSH) level as a key redox regulator in some kinds of cancer cells increases at a minimum of four times more than normal cells. This important difference in GSH concentration has been considered in designing redox-responsive DDSs [108]. Graphene-based materials can provide redox-responsive DDSs by grafting with polymers mediated with a redox-sensitive linkage such as disulfide bonds. In an effort, redox-sensitive graphene oxide nanoparticles (GON) were designed for DOX delivery in cancer treatment (Fig. 1) [109]. This system was fabricated via the redox radical polymerization of methacrylic acid (PMAA) on the PEG-modified GON (GON-PEG), subsequent PMAA crosslinking with cystamine CPMAA2-GON-PEG). The crosslinked PMAA chains prevent premature drug release while accelerating its release in the presence of a reducing agent. The release study showed that the release rate was 6-fold faster in a similar condition to tumor tissue (pH 5.0 with 10 mM GSH) than in a similar condition to normal tissue condition (pH 7.4 with 10 µM GSH). In vitro, cytotoxicity assay also presented that CPMAA2-GON-PEG had notable biocompatibility, while the DOX-loaded CPMAA2-GON-PEG demonstrated increased cytotoxicity to SiHa cells.

In another study, GO was decorated with PEG-polycaprolactone-S-S-DOX (PEG-PCL-SS-DOX) as a redox-responsive prodrug for cancer treatment [110]. To this aim, PEG-PCL-SS-DOX was first produced by DOX conjugation to the amphiphilic block copolymers PEG-PCL via a disulfide bond. Then GO was added to this system via the π-π /hydrophobic interaction with the hydrophobic block of PEG-PCL-SS-DOX. It was suggested that PEG-PCL-SS-DOX could improve the stabilization of GO dispersion and simultaneously add a redox-sensitive property to this system. In vitro results demonstrated the effective release of DOX in similar tumor site conditions that led to enhanced accumulation in tumor cells and increase anticancer activity. Additionally, in vivo results suggested that GO/PEG-PCL-SS-DOX had a notable inhibition effect on tumor growth and decrease DOX systemic toxicity. In addition to chemotherapy, GO-based nanocomposite was investigated for redox-Responsive Chlorin e6 (Ce60 for PDT of Cholangiocarcinoma [111]. In an effort, GO was grafted to MePEG and cystamine (GO-(NSSNH2)-PEG), following the amine end group was linked to Ce6, and then its photodynamic effect was assessed with various CCA cells. In comparison with free Ce6, the Ce6-conjugated GO nanocomposite showed remarkably more Ce6 cellular uptake and produced more amount of ROS resulting in increased in vitro anticancer activity. In the animal study, the Ce6-conjugated GO nanocomposite showed redox-responsiveness release leading to more accumulation in tumor tissue and increased photodynamic effect on the tumor growth with irradiation compared to free Ce6 treatment in SNU478 cell-bearing mice model.

A redox-sensitive DDS comprising GO nanosheet functionalized with hyaluronic acid (HSG) was developed for active targeting tumor cytoplasm DOX delivery [112]. Hyaluronic acid (HA) conjugating via a redox-sensitive linkage imparts several advantages to this system namely more stability in a biological medium, high drug-loading capacity, and active tumor targeting through the HA receptor. In addition, the photothermal ability of GO led to better redox response to GSH and accelerated drug release. This study indicates that GO modification can be programmed for a multi-functional DDS with several advantages such as targeting the tumor site, enhanced accumulation in the tumor site, stimuli-triggered drug release in a controlled manner, and ultimately increasing the efficiency of chemotherapeutics along with minimal damage to normal tissues. Further examples of the redox-triggered graphene-based system was listed in Table 1.

#### Thermo-stimuli graphene-based DDS

Another approach to developing an effective smart DDS is to apply materials that respond to temperature changes. Thanks to the excellent properties of graphene-based materials and thermo-responsive polymers, the powerful multi-functional DDS can be designed for therapeutic applications [113]. In this context, poly (N-isopropylacrylamide) (PNIPAM) is one of the common thermo-sensitive polymers with a lower critical solution temperature (LCST) of 32 °C in water [114]. For this purpose, the poly(L, D-lactide)- block-poly(N-isopropylacrylamide-rand-acrylic acid) conjugated from rGO (rGO-graftPDLA-block-P(NIPAAm-rand-AAc)) was fabricated with LCST of 39 °C [115]. The DOX was loaded in this delivery system with a loading capacity of 99%. It was reported that DOX release was thermo-responsiveness as it increased in response to an increasing temperature higher than LCST. Based on the in vitro cytotoxicity, DOX-loaded rGO-g-PDLA-bP(NIPAAm-rand-AAc) systems had a more lethal effect in the HepG2 cell line compared to the free DOX. In recent study, a Janus GO nanosheets modified with PNIPAM/PCL were designed as a dual DDS [116]. This novel nano-system was able to load simultaneously both of the hydrophobic (quercetin) and hydrophilic (5-FU) anti-cancer agents with a thermos-responsive release.

**Fig. 2:**
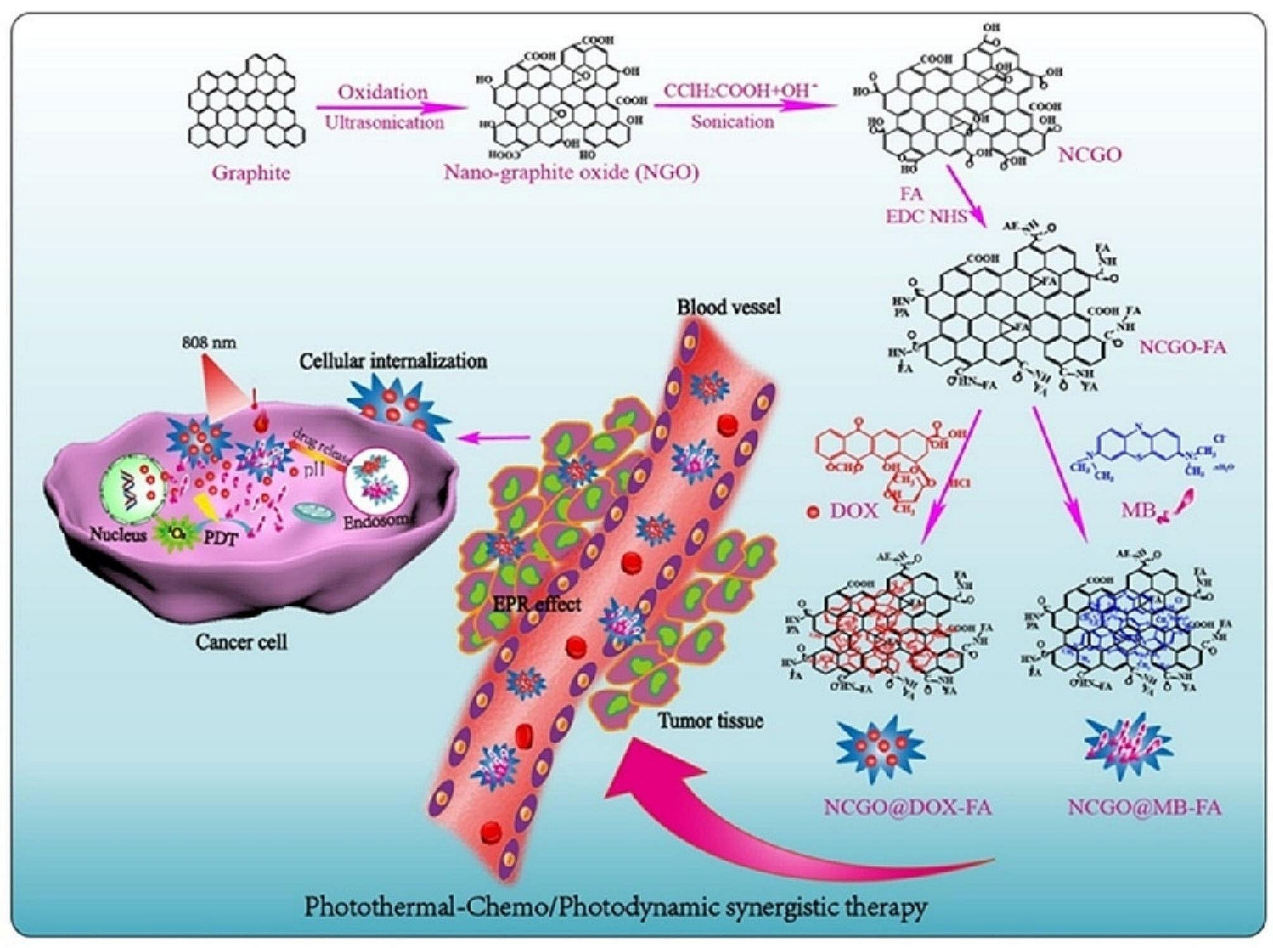
Schematic Illustrating the Fabrication of the NCGO@DOX-FA as pH and Thermal Dual-Responsive Targeted DDS for Photothermal-Chemo/Photodynamic Synergetic Therapies [117]. Note: Copyright ©2019. ACS

In the over mentioned studies, thermos-responsive graphene-based DDS was provided via GO modification with thermo-responsive polymers. Apart from this strategy, GO photothermal property was also used to development of a nanoplatform with pH/Thermal Dual-responsive drug release, tumor-targeting, and synergistic therapeutic simultaneously [117]. To this aim, a targeted nanocomplexes of GO modified with FA (NCGOFA) was developed and loaded with DOX (NCGO@DOX-FA) (Fig. 2). The results of this study demonstrated that the NCGO-FA nanocomplexes have a high-load drug capacity, targeting specificity, photostability, and photothermal conversion efficiency leading to triggered drug release by heat and in acidic conditions. Furthermore, this dual-responsive DDS exhibits a notable synergistic anticancer effect compared with individually applied chemotherapy, PTT, or PDT, or chemotherapy.

**Fig. 3:**
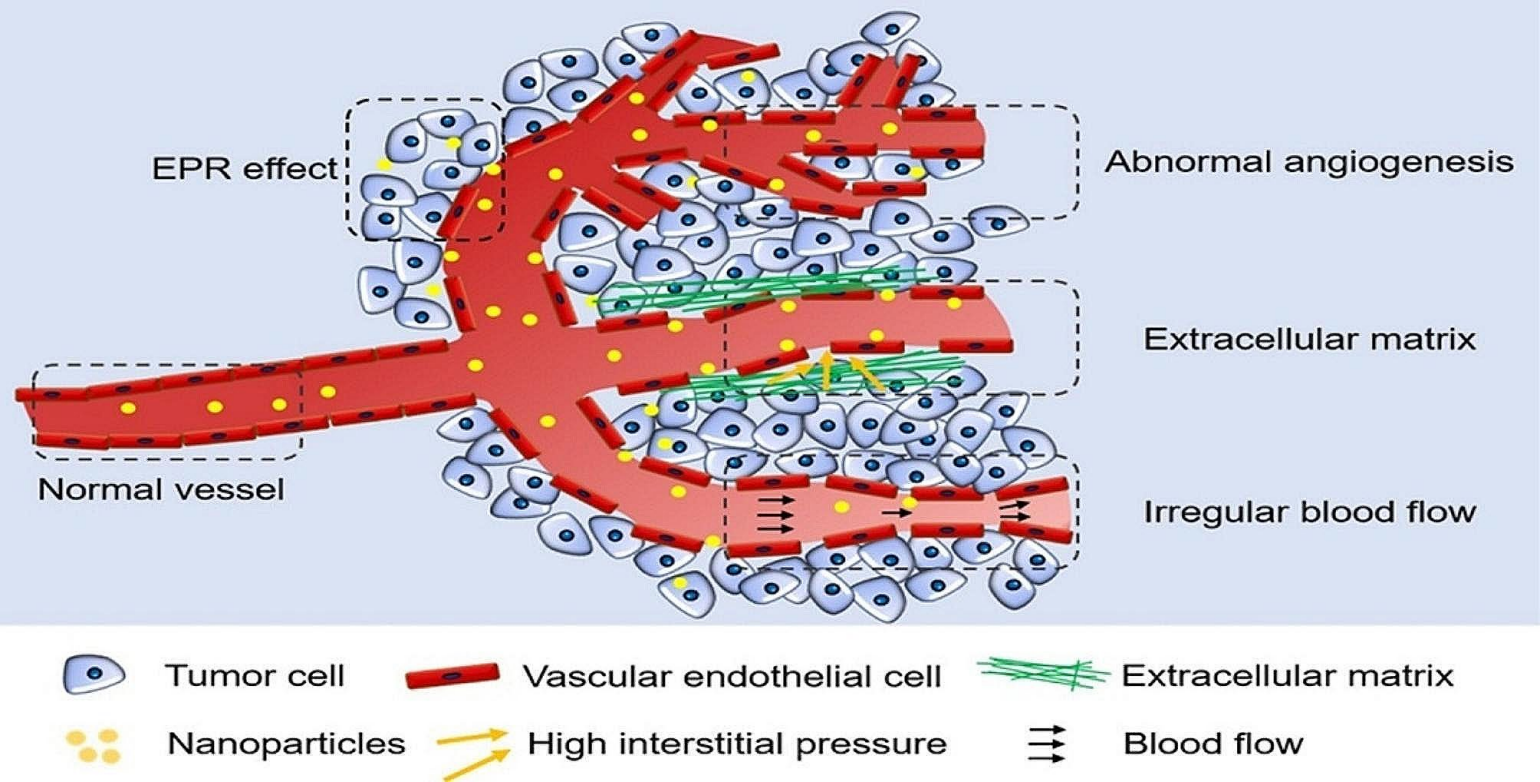
Increase the enhanced permeability and retention of effect nanodrug delivery systems in modulating tumor vessels [118]. Note: Copyright ©2021. MPOI

Nanodrugs with high molecular weight and larger particle sizes (10–500 nm)have many advantages such as: high drug loading, specific targeting, and the ability to protect the cargo from degradation and release the drug in a controlled or sustained manner. Their leak from blood vessels are more slowly than most chemotherapy drugs, which allows access to tumor tissue by leaky tumor vasculature, which is a major feature of solid tumor vasculature [118]. Nano drugs remain in the tumor bed due to the reduction of lymphatic drainage. This phenomenon has been named as the “enhanced permeability and retention” (EPR) effect to enhance the delivery of nanodrugs in tumors. Nanodrug delivery is based on drug accumulation in tumors due to the EPR effect and subsequent release. To enhance this effect in tumors, however, high interstitial fluid pressure (IFP), dense extracellular matrix (ECM), and blood vessels can be used. Tumor blood clot or embolization indicated. If the EPR effect is insufficient, the drug may be released and introduce more toxicity to normal tissues. Therefore, there is an urgent need to identify the physiological barriers that affect the EPR effect of tumors. Macromolecules between 10 and 500 nm (e.g., macromolecular anticancer agent, albumin, immunoglobulin, micelles, liposomes and protein-polymer conjugates, protein and carbon nanostructures) can selectively leak from the vascular bed and accumulate in the interstitial space. to find Tumors show different EPR effects regardless of type and size, patients or growth stages. Tumors with high blood vessel density (such as hepatocellular carcinoma) show a strong EPR effect, while tumors with low vascular density (such as pancreatic cancer) show a weak EPR effect. Nanomedicines based on EPR effect are promising to improve the effectiveness of treatment with systemic anticancer drugs. Figure 3 shows several pharmacological strategies for vascular regulation.

#### Magnetic graphene-based DDS

**Fig. 4:**
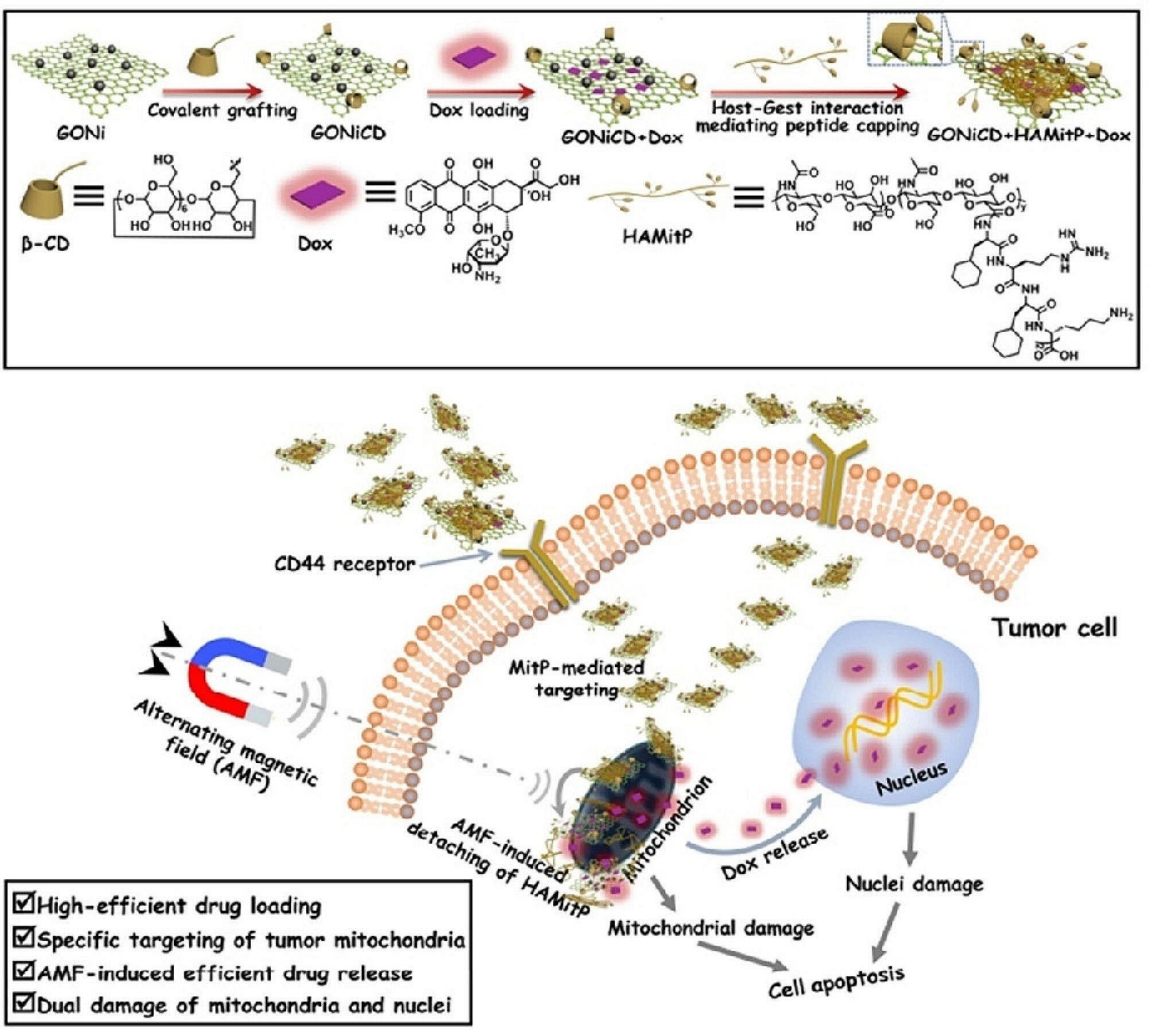
Schematic Illustration of construction of the AMF-sensitive multi-component DDS for inducing tumor cell apoptosis [119]. Note: Copyright ©2020. Wiley. CH

Recently, the combination design of graphene-based materials with magnetic nanoparticle modifications has revealed notable performance for targeted drug delivery, photothermal lethal effect on tumor cells, and magnetic-responsive drug release [7]. In magnetic-sensitive DDS, magnetic NPs (e.g., Fe, Co, Mn, or Ni derivatives) generate heating energy to increase temperature under the alternating magnetic field that leads to drug release. In an effort, a magnetic-responsive DDS was designed for DOX delivery in cancer therapy. This system was constructed of β-cyclodextrin (β-CD)/nickel NP-modified GO (GONiCD) and mitochondrial ion-targeting peptide (MitP)-conjugated HA (HAMitP) [119]. The (GONiCD) attachment to the (HAMitP) was based on host-guest interaction between β-CD and the cyclohexyl groups on MitP that lead to forming of a supramolecular assembly (Fig. 4). This multi-component DDS could enhance the DOX-loading capacity with a stimuli-controllable release in response to alternating magnetic fields and target the tumor cell mitochondria, leading to cell apoptosis via damaging both the mitochondria and the nuclei.

**Fig. 5:**
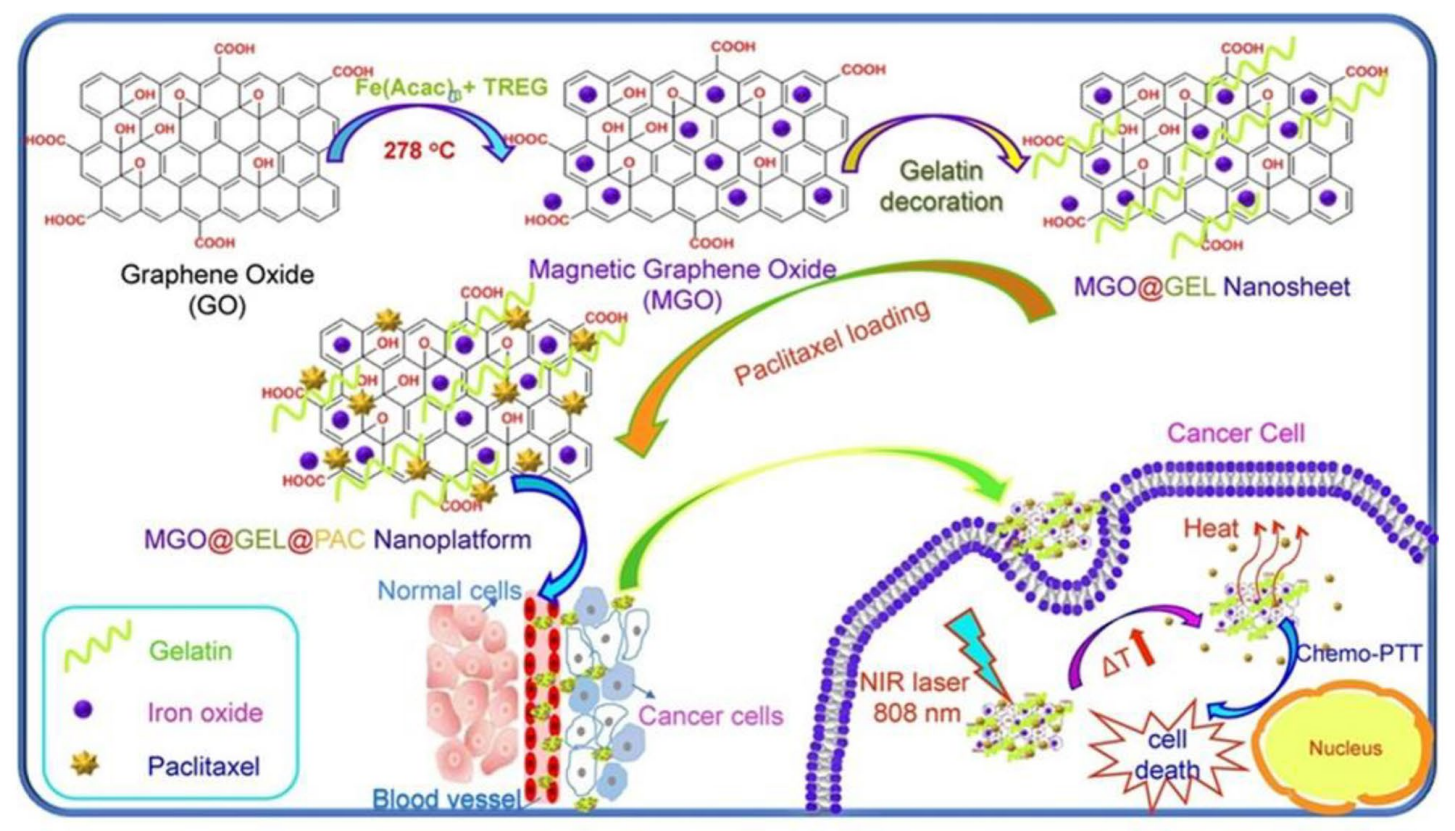
Schematic illustration of fabrication and drug delivery performance of MGO@GEL@PAC nanosheets [122]. Note: Copyright ©2021. Elsevier

In addition, magnetic NPs are used to accumulate and locally deliver drugs through a magnetic external field generated by a magnet located near the chosen treatment site. Therefore, combining magnetic NPs with graphene-based materials can indirectly enhance their therapeutic efficiency by increasing the accumulation of DDS in the desired site or synergistically enhancing the lethal effect of magnetic hyperthermia effect [89, 120, 121]. For example, PEGylated Magnetic GO (MG–NH_2_–PEG) complex was investigated at magnetically targeted DOX delivery for cancer chemotherapy and PTT. Recently, fabricated gelatin-decorated magnetic GO (MGO@GEL) nanosheet was used as pH-sensitive DDS for paclitaxel (PAC) delivery and PTT (Fig. 5) [122]. In vitro cytotoxicity assessment showed the MGO@GEL nanosheets had an acceptable biocompatibility while MGO@GEL@PAC nanosheets displayed high lethal effect especially with NIR laser. Indeed, MGO@GEL nanosheet provide PAC at the tumor site, enhance its penetration into cancer cells, and a combinational effect of chemotherapy and PTT for cancer treatment.

In one study, nanoscale GO (nGO)-Fe3O4 nanocomposite functionalized with FA was used for simultaneous tumor imaging and targeted DOX delivery [123]. In vitro results revealed that the drug release profile was pH-sensitive and FA-Fe_3_O_4_@nGO NPs were selectively uptake via the FA receptor by MGC-803 cells and had the selective anti-cancer effect. Furthermore, in vivo analysis confirmed that this nanocomposite possessed highly effective performance for in vivo MR imaging along with the selective anticancer activity.

In addition to the mentioned advantages, magnetic NPs decorated on graphene-based structures could be applied for Multimodal Bioimaging such as MRI contrast agents. In this context, Gd^3+^ ions-decorated rGO (Gd-rGO) nanosheets were studied for fluorouracil delivery as well as a contrast agent for swept-source optical coherence tomography (Swept-source OCT) and MRI [124]. Gd-rGO nanosheets presented notable contrast in comparison to bare GO for Swept-source OCT. At a magnetic field of 1.5 T, the Gd-rGO longitudinal relaxivity rate was 4 times more than the commercial contrast agent Magnevist. In the same way, GO-Fe_3_O_4_ was used as a biocompatible magnetic delivery system and MRI contrast agent [125]. DOX was non-covalently loaded on GO- Fe_3_O_4_ with 2.5-fold enhanced efficacy. Due to superparamagnetic properties, Fe_3_O_4_ grafting to GO provided magnetic targeting delivery and make contrast for MRI. These studies suggested that magnetic NPs in combined with graphene-based structures provide a novel multifunctional platform for magnetically targeted delivery, high-efficacy drug loading, and superparamagnetic properties for cancer detection.

### Light-responding in graphene-based DDS

Due to minimal body absorbance and high penetration to body tissues, NIR irradiation has been verified for photothermal anti-cancer therapy [55]. In recent years, NIR irradiation has been also investigated for photothermal drug release in nanotechnology. In this context, graphene-based nanomaterials are prevalent agents in NIR-responsive DDS due to their good ability in NIR absorbance and convert it to thermal energy. Light-responsive graphene-based DDS is generally achieved through their photothermal properties. As exposed to the optical trigger, these materials convert light to heat resulting in enhancing local temperature would lead to drug release or ablation of cancer cells [126]. On the other hand, graphene-based materials provide a platform for loading photosensitizers utilized for photodynamic cancer therapy. In these systems, photosensitizers can absorb the optical irradiation by suitable wavelengths to produce the ROS that induces apoptotic or necrotic cell death [127, 128].

#### NIR-responsive graphene-based DDS

As mentioned above, graphene-based materials present promising NIR-responsive platforms for controlled release applications due to their high efficiency in NIR irradiation to heat transformation and good biocompatibility. For example, a photothermal-responsive cytosolic DDS was developed by modification of rGO with PEG and branched polyethyleneimine (BPEI) (PEG-BPEI-rGO) for DOX delivery into cancer cells [129]. Upon photothermal induction, the endosome disruption occurred and the drug was released by NIR radiation. In addition, DOX was released more rapidly in the presence of GHS due to the weakening of its non-covalent interactions with rGO. An innovative NIR-controlled release DDS was fabricated from GO composite microcapsules that were ruptured by local heating induced by NIR-laser irradiation which consequently trigger the release of the encapsulated chemotherapeutics from these capsules [130]. GO sheets conjugated to aptamer AS1411 were loaded with berberine 9-O-pyrazole alkyl derivative with anti-cancer activity [131]. It was found that GO could help the cargo release as well as the photothermal cytotoxicity due to photothermal properties. Moreover, combining PTT and chemotherapy improved anticancer activity that can provide a promising treatment for tumors. The modified graphene derivatives 2D sheets have been applied for NIR-responsive GO-based 3D hydrogel preparation through non-covalent interactions namely π − π stacking, hydrophobic interactions, and the host − guest inclusion interactions. A NIR and pH-responsive hydrogel constructed of carboxymethyl chitosan-grafted rGO/aldehyde modified poly (ethylene glycol) (CMC-rGO/CHO-PEG) was developed for DOX delivery [132]. This nanocomposite hydrogel showed excellent potential for combined chemo-PTT with a controllable anticancer release profile.

In another study, a NIR-responsive GO-based hydrogel was fabricated of prodrug-conjugated GO and α-cyclodextrin (α-CD) for both hydrophilic and hydrophobic drug delivery [133]. To this aim, GO was first modified with camptothecin-PEG (CPT-PEG) via the non-covalent interactions between GO sheets and CPT moieties. Then, α-CD was added to the GO-CPT-PEG structure by the host-guest inclusion interactions between α-CD and PEG, and the hydrogel was formed. NEXT, the GO-based hydrogel was used for loading 5-FU as a hydrophilic chemotherapeutic. This GO-based hydrogel showed a gel-sol phase transition triggered by NIR irradiation that led to the release of loaded drug. In addition, in vivo study revealed that the 5-FU loaded GO-CPT-PEG/α-CD hydrogel as a dual-drug carrier has promising potential for combination therapy. In the same way, NIR, temperature, and pH-responsive GO-based hydrogel were constructed for 5-FU delivery with a controllable release profile [134]. In this structure, GO sheets act not only as a core material for conjugating to the pH-sensitive polymer and additional cross-linking but also trigger the gel − sol transition of hydrogel via NIR light to heat conversion. It was suggested that this triple-responsive GO-based hydrogel could be beneficial for the controlled release of drugs and would be a good candidate in the field of DDS.

#### PTT

Thanks to the NIR to heat conversion ability, graphene-based nanomaterials have been widely used for the delivery of anti-cancer agents and PTT simultaneously leading to a synergistic effect in cancer treatment [11]. It was reported that rGO combined with the 980-nm laser irradiation showed an ideal thermal lethal effect in pancreatic cancer treatment in a mouse model [135]. The experimental data obtained from this study indicated that rGO photothermal effect depended on its concentration and laser dose. Fabricated DOX-loaded bovine serum albumin (BSA)-conjugated rGO (DOX-BSA-rGO) nanosheets were used for chemo-photothermal cancer treatment [136]. In vivo study exhibited that DOX-BSA-rGO nanosheets were uptake in a dose-dependence manner with acceptable biocompatibility and synergic chemo-photothermal effect on brain tumor cells. In another study, FA-GO nanosheets modified MNO2 NPs was employed for targeted PTT and MR imaging [137]. In addition to PTT, MnO_2_ NPs can degrade the H_2_O_2_ in the tumor cell microenvironment and counter hypoxia. FA-GO nanosheets modified MNO_2_ NPs were employed for targeted PTT and MR imaging. This nanocomposite was uptake by the Hela cells overexpressing FA receptors, triggered the NIR-light mediated hyperthermia. GO nanocomposite loaded with PEGylated SPION-grafted methotrexate (GO-SPION-MTX) was developed for PTT and chemotherapy in breast cancer [138]. It was found that these nanocomposites were uptake by the folate-receptor-positive tumor cells and demonstrated high cytotoxicity resulting from the combination of chemo-PTT.

**Fig. 6:**
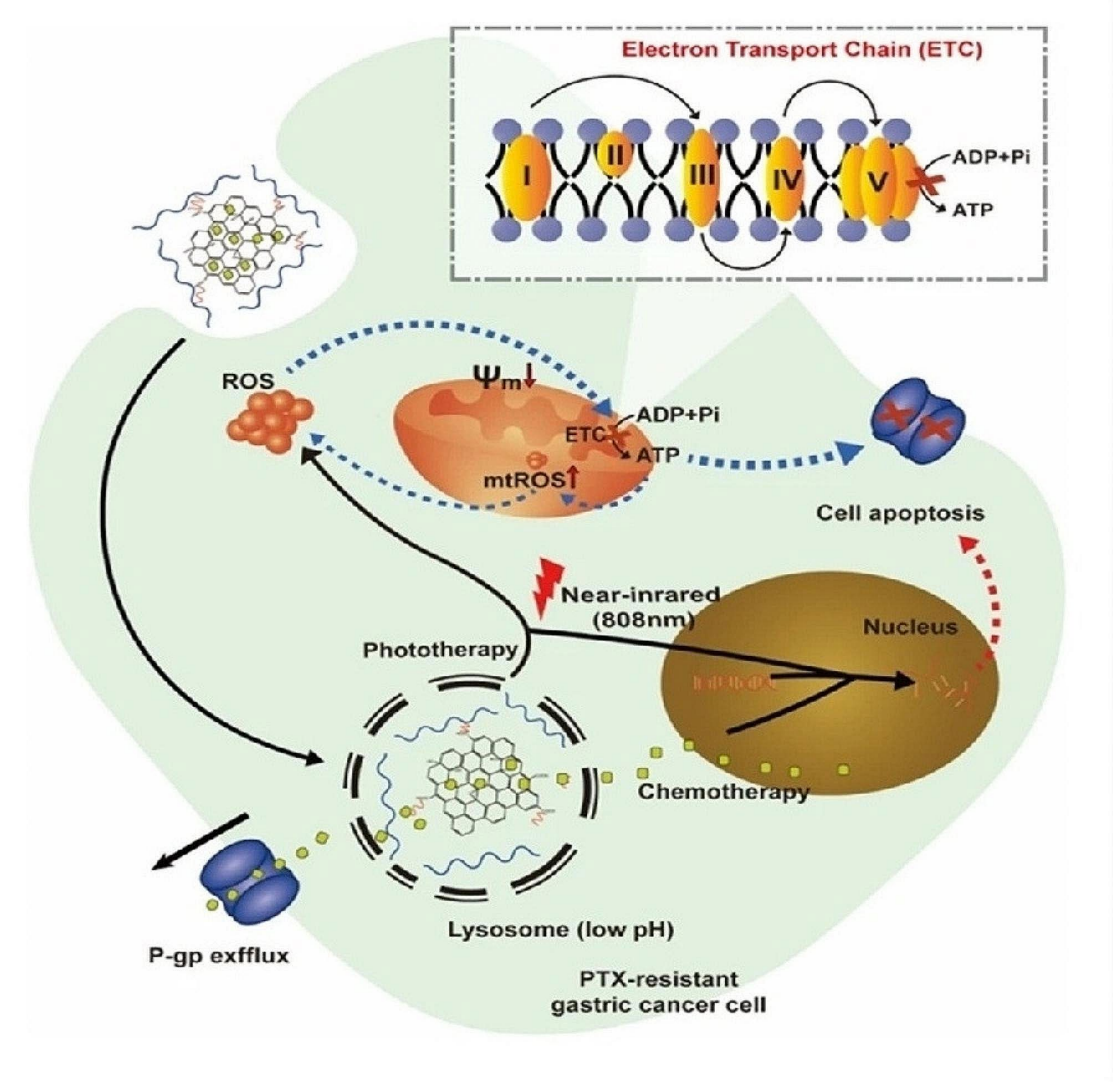
Schematic illustration of PTX@GO-PEG-OSA performance as a desirable strategy with dual pH and thermal-responsive drug release for chemo/photothermal/photodynamic therapy in reverse PTX’s resistance [70]. Note: Copyright ©2021. BMC

Recently, a G-based nanocomposite constructed of GO-PEG-modified oxidized sodium alginate (OSA) nanosheets (GO-PEG-OSA) were developed for the treatment of PTX-resistant gastric cancer (Fig. 6) [70]. In comparison with free drugs, PTX@GO-PEG-OSA showed more lethal effects on gastric cancer due to possessing thermal and pH-responsive drug release properties. Additionally, upon NIR irradiation PTX@GO-PEG-OSA could generate ROS that induced mitochondrial damage leading to suppressing the out-pumping function of P-glycoprotein and ultimately reversing PTX-resistant in gastric cancer cells. Therefore, PTX@GO-PEG-OSA with combining chemotherapy/PTT/PDT can be considered an effective strategy to reverse PTX’s resistance.

Although the graphene-based materials showed promising potential for in vivo PTT, their self-aggregation and accumulation in the target site remain challenging. To address these challenges, poloxamer-modified rGOs (prGOs)- loaded in human mesenchymal stem cells (hMSCs) were developed for targeted PTT [139]. pRGOs did not demonstrate any self-aggregation compared to rGOs. In vivo results revealed that pRGOs-loaded hMSCs remarkably improved tumor-targeting efficiency and generated higher heat than bare rGOs under laser irradiation.

**Fig. 7:**
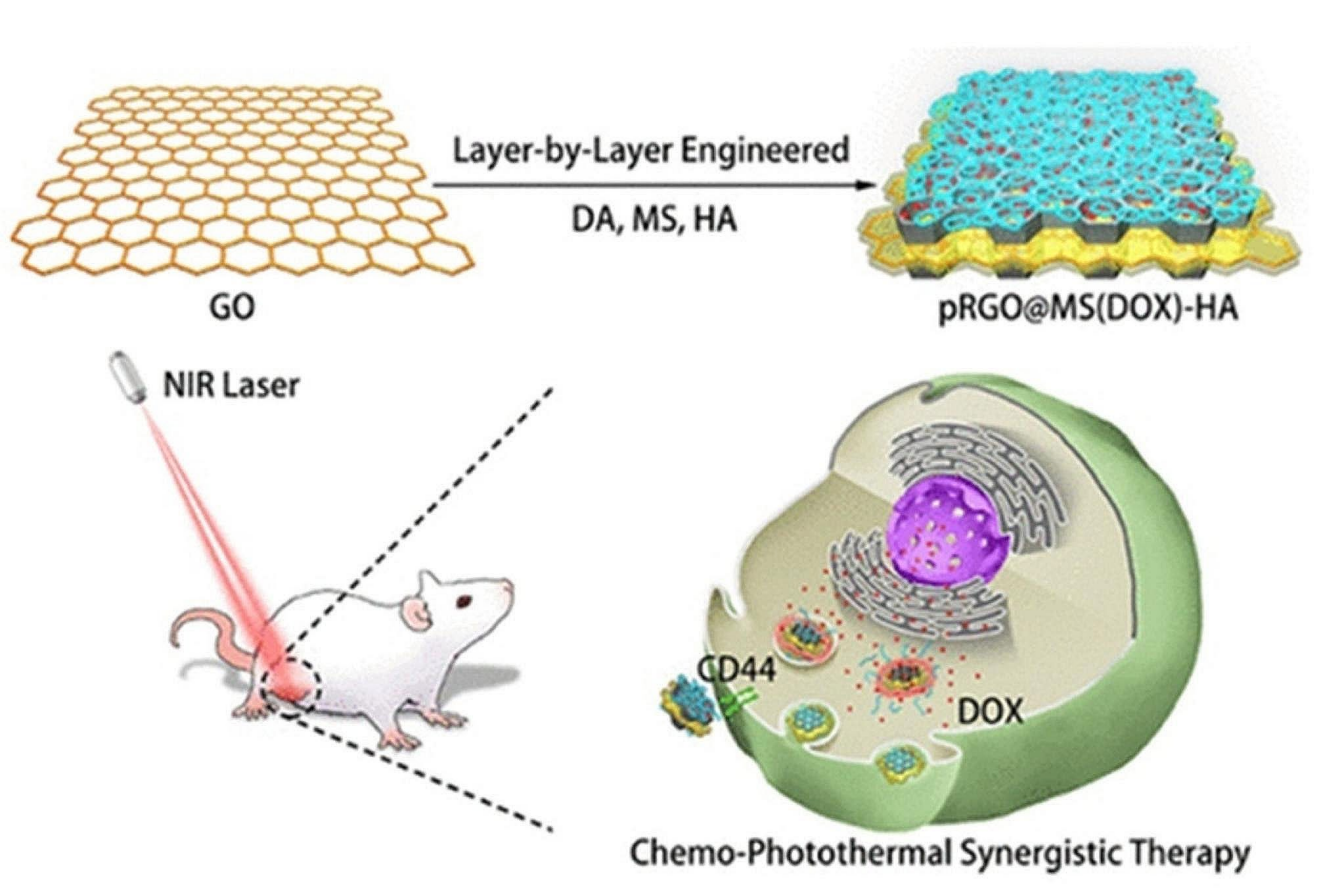
Schematic illustration of multifunctional pRGO@MS(DOX)-HA for targeted chemo-PTT in cancer treatment [140]. Note: Copyright ©2017. ACS

The integration of various nanomaterials into a unique platform lets to bring their advantages together in a synergistic manner in cancer treatment. For example, polydopamine-modified rGO was coated with mesoporous silica (MS) and further conjugated with HA (pRGO@MS-HA) and used as a multifunctional nano-platform for targeted chemo-PTT in cancer treatment (Fig. 7) [140]. In addition to heat-generating of rGO, polydopamine modification could enhance rGO biocompatibility while MS employed for DOX loading, and HA acted as a targeting ligand. It was reported that DOX release was pH and NIR-responsive, which could improve the efficiency of this system. In vitro results revealed that pRGO@MS(DOX)-HA showed excellent photothermal effect along with a notable lethal effect on tumor cells with good specificity toward target cells. In vivo study confirmed that pRGO@MS(DOX)-HA possessed a synergistic antitumor effect more than individual chemo or photothermal effect.

In the same way, a targeted dual pH/thermal-responsive DDS with chemo-photothermal effect was fabricated by integrating a DNA aptamer with polydopamine-rGO nanosheets (rGO-PDA) nanosheets [141]. The rGO-PDA nanosheets simultaneously possessed the photothermal properties to generate hyperthermia, served as a nano-carrier for DOX loading. In addition, DNA aptamer modification provided a supplementary carrier for drug loading, served as a targeting ligand to recognize specific cell, and was able to control DOX release. All these advantages led to achieve an effective multifunctional DDS for chemo-PTT in a synergistic manner.

In recent years, graphene-based materials have been employed to construct hydrogels for achieving a multimodal DDS. For instance, GO-hybridized nanogels were developed to deliver chemotherapeutic agents and simultaneously presented PTT against cancer cells [142]. These nanogels were obtained by in situ combining GO nanosheets into alginate through a double emulsion method using disulfide bonds as crosslinkers. In the following, DOX was loaded by electrostatic interactions. The controllability of the PH/redox-sensitive DOX release improved the DOX uptake and long-term accumulation in cancer cells which along with GO photothermal property, led to an excellent anticancer effect, representing their potential for anticancer combination therapy. Additionally, the GO photothermal effect was employed for controlling drug release and PTT simultaneously. For example, a targeted light-responsive DDS was developed by a Cy5.5-AS1411 aptamer-conjugated GO wrapping on the surface of the DOX-loaded mesoporous silica NPS (MSN-Dox@GO-Apt) [143]. In this system, GO acts as a gatekeeper to control the Dox release in response to NIR irradiation and convert NIR to heat to induce PTT. The results showed that a combination of chemotherapy and PTT in a single platform resulted in a synergic effect much more effective than monotherapies, which can be considered a new strategy for cancer treatment.

#### PDT

PDT is a therapeutic modality employed with the purpose of eradicating malignant cells within tumors that involves the utilization of light with a certain wavelength to initiate the activation of molecules known as photosensitizers (PS). Subsequently, these molecules generate reactive oxygen species (ROSs) that possess the ability to induce apoptosis in the tumor cells [144]. In order to optimize the targeted delivery of PDT agents to tumor sites by reducing the doses to be effective, allowing easy cell entrance, and reducing skin sensitivity to light, the utilization of nanocarriers is imperative [145]. In recent times, researchers have conducted investigations on the optical loading properties of G, with a particular focus on its potential applications in the medical domain. Notably, G has been recognized as a crucial element in the development of miniature technological platforms for healthcare purposes. The exploration of theranostics started by employing G-based materials as carriers for both therapeutic substances and imaging agents [146]. Consequently, this development prompted subsequent investigations into the utilization of nanotechnology-based PDT for the purpose of achieving more targeted and precise cancer treatment. G has the capability to effectively catch light inside the NIR band. This enables the investigation of its potential application in cancer treatment by the utilization of light therapy, both internally and externally [147].

Researchers are trying to find more efficient methods of treating cancer. They are looking at how the nanoplatforms may complement existing treatments to better handle cancer as presented in Table 1. Using π-π stacking of GO, Tian’s group used PEG-GO and Ce6 as PS. The chemical was taken up by the cervical cancer cells, and when exposed to light, it triggered the generation of ROS. When compared to Ce6, GO-PEG-Ce6 proved more successful in the therapy of cancer [128]. GO was proposed by Huang and Collagenous as a Ce6 carrier. Ce6 was bound to folic acid-functionalized GO through π-π stacking binding, which is consistent with prior research. Researchers showed that when exposed to radiation, stomach cancer cells were killed by the system [148]. Zhou and coworkers used π-π stacking interaction to successfully add hypocrellin B (HB) as a PS to GO. When subjected to radiation, they showed that the material produced ROSs [127, 149]. Hypocrellin A and 7-ethyl-10-hydroxycamptothecin were combined on GO for this study. More lung cancer cells died when this mixture was exposed to light. This demonstrates the synergistic benefits of combining chemotherapy with PDT [149]. Increasing cellular sensitivity to ROS by inhibiting the DNA oxidative damage repair enzyme MTH1 may increase the efficacy of PDT. In order to combine PDT with DOX, Huang and coworkers used a GO-based nanocarrier to transport TH287 (an MTH1 inhibitor) and DOX to cancer cells [150]. They grafted PEG, FA, the photosensitizer indocyanine green (ICG), TH287, and DOX onto the GO nanocarrier. Proliferation and migration were inhibited, and endoplasmic reticulum (ER) stress-induced apoptosis and autophagy were augmented, in MNNG/HOS, MG63, U2OS, and SaOS-2 (osteosarcoma cancer) cells thanks to the efficient transport of DOX and TH287 with the PEG-GO-FA/ICG carrier. The distinctive characteristics of G have enhanced the efficacy and expediency of cancer treatment when utilizing G-based nanocarriers for the delivery of PDT and chemotherapy medications, surpassing the individual effectiveness of these methods.

Recently, G-derived nanocarriers with triple capacities have been conceived and produced to increase the efficacy of cancer therapy approaches. The development of a GO-based nanocarrier that is pH- and temperature-sensitive makes cancer treatment that combines PTT, PDT, and chemotherapy easier. Nanocomplexes GO (NCGO) NCGO@DOX-FA, and NCGO@methylene-FA are complexes formed when GO is grafted with the medicines DOX and methylene blue, respectively. This nanocarrier is distinguished from others in a number of ways, including its large surface area, photostability, and capacity to transport medications to specific locations. The FA receptors effectively facilitate the targeted delivery of the complexes into the cancer cells, leading to the subsequent release of the medication triggered by either the acidic pH or heat conditions [117]. A subsequent investigation by Ding et al. resulted in the development of a GO-involved nanoparticle with the specific goal of targeting cancer [151]. The nanoparticle was noncovalently functionalized with cucurbit [7] uril (CB [7]) in order to serve as a potential agent for drug delivery. Hence, the NGO was painted with carbon black (CB) and became known as GO-CB. Chlorin e6, a PS, and AQ4N (banoxantrone dihydrochloride), a hypoxia-responsive prodrug, were subsequently included in this composite material to alter its properties. Next, we added a CB [7] (more precisely, oxaliplatin, abbreviated as OX) guest molecule and a CD44-targeting chemical, ADA-hyaluronic acid (ADA-HA), that is well-known for improving biocompatibility. In the presence of OX and AQ4N, this nanoplatform has the potential to act as a PTT-PDT agent for the treatment of L02 (human fetal hepatocyte line) and B16 (murine melanoma) cells. This research demonstrates a drug delivery platform that has the potential to be used in a variety of different ways for cancer therapy, both in vivo and in vitro. In summary, it can be inferred that the combination of PDT or PTT with chemotherapy has the potential to successfully eliminate cancer cells through the utilization of graphene’s natural NIR absorption properties. The utilization of G-based vehicles has considerable potential in the field of cancer treatment.

## Graphene-based hybrid composites biocompatibility

The use of biomaterials based on G in the fields of in vivo biomedicine and cancer therapy has always been a subject of debate. The desired toxicity of G derivatives towards bacteria and cancer cells is tempered by concerns regarding their potential toxicity as well as the limited understanding of their metabolism and long-term effects on different cell types, tissues, and organs. Consequently, the utilization of these derivatives in the field of biomedicine may face significant limitations. Interactions between cell membranes and G-based nanomaterials can potentially lead to negative consequences, such as the degradation of the cellular membrane and cytotoxicity [152, 153]. The cell membrane phospholipids are composed of a phosphate moiety that is linked to two fatty acyl chains. The main head groups include choline, serine, glycerol, ethanolamine, inositol, and phosphatic acid. The presence of multiple head groups with different properties gives phospholipids their unique and identifiable characteristics. In addition, cellular membranes contain cholesterol molecules that have important functions in preserving the structure of the membrane, keeping it fluid, and regulating the activities of proteins associated with it [154]. G that is pure and lacks charges on its basal plane is unable to engage in electrostatic interactions with phospholipids. However, it shows a tendency to interact with the lipid tails through hydrophobic interactions. Furthermore, the interactions between the G backbone and the cholesterol residue have the ability to extract or remove cholesterol molecules from the cellular membrane. This process can result in membrane impairment [153].

In addition, it has been discovered that lipid membrane and nanoparticle interactions extend beyond simple surface contacts. G-based nanomaterials can enter the cytoplasm due to their small size and sharp edges. It has been shown that these chemicals may effectively permeate the cell membrane, having adverse effects on the membrane and resulting in the release of intracellular contents. Once inside living cells, they can cause harm by releasing harmful ROS [153]. Because ROS have the ability to decrease mitochondrial membrane potential (MMP), they can cause mitochondrial dysfunction. Lactate dehydrogenase (LDH) is released as a result of their presence, which might cause damage to cell membranes. Unsaturated fatty acids found in membrane lipids can react with ROS to start lipid peroxidation. Lipid peroxides, such as malondialdehyde (MDA), are produced during this process. According to the results, GO can cause the release of ROS outside and inside cells, even at low concentrations. The magnitude and duration of this impact are dose- and exposure-dependant. Thus, ROS generation, mitochondrial malfunction, and the subsequent release of LDH are the primary mechanisms associated with cellular death [154]. Recent studies on the toxicity and physiological impacts of G nanoparticles have shown conflicting results, suggesting that even subtle alterations to their structure can significantly alter their properties.

Numerous studies, using a variety of animal delivery modalities, have established the biodistribution and in vivo toxicology of G and its functionalized derivatives. Research by Liu et al. found that the toxicity and biodistribution of GO varied with both dose and particle size. Primary deposition of GO was seen in the liver, with only trace aggregates in the lungs and spleen. When comparing the effects of GO particles of varying sizes, it was found that those of intermediate size accumulated more heavily in the lungs. In addition, after 180 min, almost 19% of the intermediate-sized GO was still present, demonstrating its potential for long-term residency and sustained impact [155]. According to studies, even trace amounts of GO can enter the circulation and cause damage to vital organs like the liver, brain, kidneys, and lungs [156–158 and 159]. Studies show that GO can cross the placental and blood-brain barriers and cause harm to the developing embryo. Some research has shown that PEG-functionalized GQDs and GO derivatives are not absorbed into the bloodstream and are instead eliminated in the stool. Ultimately, several investigations suggest that G-based nanomaterials exhibit a degree of safety for biological applications. However, more recent examinations focusing on toxicity have cast doubt on this assertion. The reliability of in vivo outcomes may be compromised due to potential inconsistencies in material usage or the duration of investigations. Therefore, it is necessary to conduct more research in accordance with international rules to investigate the safety of these remarkable nanoparticles for medical applications.

## Conclusion

The rising cancer death rate and demand for cancer treatments have prompted the everyday development of new cancer diagnoses and therapies. Researchers have recently produced G derivative-based cancer diagnosis and treatments. G derivatives may solve viral vectors’ carcinogenic and immunogenic concerns as gene and drug delivery platforms for cancer treatment. Graphene’s large surface area, stability, optical and photoluminescent characteristics, and easy, cost-effective functionalization give it this promise. In addition to their noteworthy properties, G-based nanomaterials have hurdles that must be overcome for biomedical use. Additional imaging and integrated treatment aspects may be attained with the use of G-based nanocomposites. It is suggested that tumor ligands that bind to G-based nanocomposites boost their specific or targeted medication delivery capability. G-based nanocomposites with a functionalized or coated surface may be used as a nanoplatform for cancer diagnosis and therapy. Since G-based nanocomposites exposure can lead to major health issues, investigating their biocompatibility is crucial.

## Data Availability

The authors confirm that the data supporting the finding of this study are available in the article.

## References

[1] Alimardani V, Farahavar G, Salehi S, Taghizadeh S, Ghiasi MR, Abolmaali SS (2021). Gold nanocages in cancer diagnosis, therapy, and theranostics: a brief review. Front Mater Sci.

[2] Alimardani V, Abolmaali SS, Tamaddon AM, Ashfaq M (2021). Recent advances on microneedle arrays-mediated technology in cancer diagnosis and therapy. Drug Delivery Translational Res.

[3] Alimardani V, Abolmaali SS, Yousefi G, Nowroozzadeh MH, Tamaddon AM (2023). In-situ nanomicelle forming microneedles of poly NIPAAm-b-poly glutamic acid for trans-scleral delivery of dexamethasone. J Ind Eng Chem.

[4] Alimardani V, Rahiminezhad Z, DehghanKhold M, Farahavar G, Jafari M, Abedi M, Moradi L, Niroumand U, Ashfaq M, Abolmaali SS (2023). Nanotechnology-based cell-mediated delivery systems for cancer therapy and diagnosis. Drug Delivery Translational Res.

[5] Gani IH, Al-Obaidi Z (2023). MgO NPs catalyzed the synthesis of novel pyridin-3-yl-pyrimidin-2-yl-aminophenyl-amide derivatives and evaluation of pharmacokinetic profiles and biological activity. Front Mater.

[6] Rahiminezhad Z, Tamaddon A, Dehshahri A, Borandeh S, Abolmaali SS, Najafi H, Azarpira N (2022). PLGA-graphene quantum dot nanocomposites targeted against αvβ3 integrin receptor for sorafenib delivery in angiogenesis. Biomaterials Adv.

[7] Najmi M, Ayari MA, Sadeghsalehi H, Vaferi B, Khandakar A, Chowdhury ME, Rahman T, Jawhar ZH (2022). Estimating the dissolution of anticancer drugs in supercritical carbon dioxide with a stacked machine learning model. Pharmaceutics.

[8] Kadhum WR, Al-Zuhairy SA, Mohamed MB, Abdulrahman AY, Kadhim MM, Alsadoon Z (2022). Teoh. A nanotechnological approach for enhancing the topical drug delivery by newly developed liquid crystal formulations. Int J Drug Deliv Technol.

[9] Rezaei M, Rahmani E, Khouzani SJ, Rahmannia m, Ghadirzadeh E, Bashghareh P, Chichagi F, Fard SS, Esmaeili S, Tavakoli R, Seighalani HH (2023). Role of artificial intelligence in the diagnosis and treatment of diseases. Kindle.

[10] Borandeh S, Alimardani V, Abolmaali SS, Seppälä J (2021). Graphene family nanomaterials in ocular applications: physicochemical properties and toxicity. Chem Res Toxicol.

[11] Hoseini-Ghahfarokhi M, Mirkiani S, Mozaffari N, Abdolahi Sadatlu MA, Ghasemi A, Abbaspour S, Akbarian M, Farjadian F, Karimi M (2020). Applications of Graphene and Graphene Oxide in Smart Drug/Gene Delivery: is the World still flat?. Int J Nanomed.

[12] Alimardani V, Abolmaali SS, Borandeh S (2019). Antifungal and antibacterial properties of graphene-based nanomaterials: a mini-review. J Nanostruct.

[13] Muazim K, Hussain Z (2017). Graphene oxide—A platform towards theranostics. Mater Sci Engineering: C.

[14] Hoseini-Ghahfarokhi M, Mirkiani S, Mozaffari N, Abdolahi Sadatlu MA, Ghasemi A, Abbaspour S, Akbarian M, Farjadian F, Karimi M. Applications of graphene and graphene oxide in smart drug/gene delivery: is the world still flat? Int J Nanomed, (2020) 9469–96. 10.2147/IJN.S265876 PMC7710865 33281443

[15] Fedotov А, Prischepa S, Fedotova J, Bayev V, Ronassi AA, Komissarov I, Kovalchuk N, Vorobyova S, Ivashkevich O (2020). Electrical conductivity and magnetoresistance in twisted graphene electrochemically decorated with Co particles.

[16] Moradi O, Mahdavian L (2021). Simulation and computational study of graphene oxide nano-carriers, absorption, and release of the anticancer drug of camptothecin. J Mol Model.

[17] Bhatt S, Punetha VD, Pathak R, Punetha M. Graphene in nanomedicine: a review on nano-bio factors and antibacterial activity. Colloids Surf B, (2023) 113323. 10.1016/j.colsurfb.2023.113323 37116377

[18] Yang G, Zhang R, Liang C, Zhao H, Yi X, Shen S, Yang K, Cheng L, Liu Z (2018). Manganese dioxide coated WS2@ Fe3O4/sSiO2 nanocomposites for pH-responsive MR imaging and oxygen‐elevated synergetic therapy. Small.

[19] Housman G, Byler S, Heerboth S, Lapinska K, Longacre M, Snyder N, Sarkar S (2014). Drug resistance in cancer: an overview. Cancers.

[20] Ashfaq M, Talreja N, Chauhan D, Afreen S, Sultana A, Srituravanich W (2022). Two-dimensional (2D) hybrid nanomaterials for diagnosis and treatment of cancer. J Drug Deliv Sci Technol.

[21] Wang L, Xiong Q, Xiao F, Duan H (2017). 2D nanomaterials based electrochemical biosensors for cancer diagnosis. Biosens Bioelectron.

[22] Nanda SS, Papaefthymiou GC, Yi DK (2015). Functionalization of graphene oxide and its biomedical applications. Crit Rev Solid State Mater Sci.

[23] Zhao W, Ghaznavi A, Gao Y, Wu M, Xu J (2023). Synthesis of a functionalized carbon nanotube graphene composite enabling pH-responsive electrochemical sensing for biomedical applications. ACS Appl Electron Mater.

[24] Yang Y, Asiri AM, Tang Z, Du D, Lin Y (2013). Graphene based materials for biomedical applications. Mater Today.

[25] Chen D, Feng H, Li J (2012). Graphene oxide: preparation, functionalization, and electrochemical applications. Chem Rev.

[26] Wen W, Song Y, Yan X, Zhu C, Du D, Wang S, Asiri AM, Lin Y (2018). Recent advances in emerging 2D nanomaterials for biosensing and bioimaging applications. Mater Today.

[27] Esteban-Fernández B, de Ávila E, Araque S, Campuzano M, Pedrero B, Dalkiran R, Barderas R, Villalonga E, Kiliç JM, Pingarrón (2015). Dual functional Graphene Derivative-based Electrochemical platforms for detection of the TP53 gene with single nucleotide polymorphism selectivity in Biological samples. Anal Chem.

[28] Shayan S, Osgoei LT, Jouni FJ (2023). Effect of capecitabine and melatonin on HER2+ (SK-BR-3) and HER2-(MCF7) human breast cancer cell lines. Trop J Pharm Res.

[29] Kilic T, Erdem A, Erac Y, Seydibeyoglu MO, Okur S, Ozsoz M (2015). Electrochemical Detection of a Cancer Biomarker Mir-21 in cell lysates using Graphene Modified sensors. Electroanalysis.

[30] Azimzadeh M, Rahaie M, Nasirizadeh N, Ashtari K, Naderi-Manesh H (2016). An electrochemical nanobiosensor for plasma miRNA-155, based on graphene oxide and gold nanorod, for early detection of breast cancer. Biosens Bioelectron.

[31] Tran HV, Piro B, Reisberg S, Duc HT, Pham MC (2013). Antibodies Directed to RNA/DNA hybrids: an Electrochemical Immunosensor for MicroRNAs detection using graphene-composite electrodes. Anal Chem.

[32] Rusling JF, Kumar CV, Gutkind JS, Patel V (2010). Measurement of biomarker proteins for point-of-care early detection and monitoring of cancer. Analyst.

[33] Chikkaveeraiah BV, Bhirde AA, Morgan NY, Eden HS, Chen X (2012). Electrochemical immunosensors for detection of Cancer protein biomarkers. ACS Nano.

[34] Feng L, Wu L, Wang J, Ren J, Miyoshi D, Sugimoto N, Qu X (2012). Detection of a prognostic indicator in early-stage cancer using functionalized graphene‐based peptide sensors. Adv Mater.

[35] Wei T, Tu W, Zhao B, Lan Y, Bao J, Dai Z (2014). Electrochemical monitoring of an important biomarker and target protein: VEGFR2 in cell lysates. Sci Rep.

[36] Winterbourn CC (2008). Reconciling the chemistry and biology of reactive oxygen species. Nat Chem Biol.

[37] Chang H, Wang X, Shiu K-K, Zhu Y, Wang J, Li Q, Chen B, Jiang H (2013). Layer-by-layer assembly of graphene, au and poly(toluidine blue O) films sensor for evaluation of oxidative stress of tumor cells elicited by hydrogen peroxide. Biosens Bioelectron.

[38] Xiao F, Li Y, Zan X, Liao K, Xu R, Duan H (2012). Growth of metal–metal oxide nanostructures on freestanding graphene paper for flexible biosensors. Adv Funct Mater.

[39] Nawrot W, Drzozga K, Baluta S, Cabaj J, Malecha K (2018). A fluorescent biosensors for detection vital body fluids’ agents. Sensors.

[40] He Y, Lin Y, Tang H, Pang D (2012). A graphene oxide-based fluorescent aptasensor for the turn-on detection of epithelial tumor marker mucin 1. Nanoscale.

[41] Tan J, Lai Z, Zhong L, Zhang Z, Zheng R, Su J, Huang Y, Huang P, Song H, Yang N, Zhou S, Zhao Y. A Graphene Oxide-Based Fluorescent Aptasensor for the Turn-on Detection of CCRF-CEM, Nanoscale Research Letters, 13 (2018) 66. 10.1186/s11671-017-2403-3 PMC5878827 29605867

[42] Loh KP, Bao Q, Eda G, Chhowalla M (2010). Graphene oxide as a chemically tunable platform for optical applications. Nat Chem.

[43] Lu C-H, Zhu C-L, Li J, Liu J-J, Chen X, Yang H-H (2010). Using graphene to protect DNA from cleavage during cellular delivery. Chem Commun.

[44] Cai B, Wang S, Huang L, Ning Y, Zhang Z, Zhang G-J (2014). Ultrasensitive label-free detection of PNA–DNA hybridization by reduced Graphene Oxide Field-Effect Transistor Biosensor. ACS Nano.

[45] Cai B, Huang L, Zhang H, Sun Z, Zhang Z, Zhang G-J (2015). Gold nanoparticles-decorated graphene field-effect transistor biosensor for femtomolar MicroRNA detection. Biosens Bioelectron.

[46] Hu SH, Chen YW, Hung WT, Chen IW, Chen SY (2012). Quantum-dot‐tagged reduced graphene oxide nanocomposites for bright fluorescence bioimaging and photothermal therapy monitored in situ. Adv Mater.

[47] Antwi-Boasiako AA, Dunn D, Dasary SS, Jones YK, Barnes SL, Singh AK (2017). Bioconjugated graphene oxide‐based Raman probe for selective identification of SKBR3 breast cancer cells. J Raman Spectrosc.

[48] Dojčilović R, Pajović JD, Božanić DK, Jović N, Pavlović VP, Pavlović VB, Acković LL, Zeković I, Dramićanin M, Kaščaková S, Réfrégiers M, Rašić G, Vlahović B, Djoković V (2018). 2D Mater.

[49] Younis MR, He G, Lin J, Huang P. Recent advances on Graphene Quantum Dots for Bioimaging Applications. Front Chem, 8 (2020). 10.3389/fchem.2020.00424 PMC7283876 32582629

[50] McCallion C, Burthem J, Rees-Unwin K, Golovanov A, Pluen A (2016). Graphene in therapeutics delivery: problems, solutions and future opportunities. Eur J Pharm Biopharm.

[51] Yang K, Wan J, Zhang S, Tian B, Zhang Y, Liu Z (2012). The influence of surface chemistry and size of nanoscale graphene oxide on photothermal therapy of cancer using ultra-low laser power. Biomaterials.

[52] Dasari Shareena TP, McShan D, Dasmahapatra AK, Tchounwou PB (2018). A review on graphene-based nanomaterials in biomedical applications and risks in environment and health. Nano-micro Lett.

[53] Wu H, Lu W, Shao J-J, Zhang C, Wu M-B, Li B-H, Yang Q-h (2014). Ph-dependent size, surface chemistry and electrochemical properties of graphene oxide. Carbon.

[54] Muthoosamy K, Abubakar IB, Bai RG, Loh H-S, Manickam S (2016). Exceedingly higher co-loading of Curcumin and Paclitaxel onto Polymer-functionalized reduced Graphene Oxide for highly potent synergistic Anticancer treatment. Sci Rep.

[55] Liu J, Cui L, Losic D (2013). Graphene and graphene oxide as new nanocarriers for drug delivery applications. Acta Biomater.

[56] Liu Z, Robinson JT, Sun X, Dai H (2008). PEGylated nanographene oxide for delivery of water-insoluble cancer drugs. J Am Chem Soc.

[57] Sahoo NG, Bao H, Pan Y, Pal M, Kakran M, Cheng HKF, Li L, Tan LP (2011). Functionalized carbon nanomaterials as nanocarriers for loading and delivery of a poorly water-soluble anticancer drug: a comparative study. Chem Commun.

[58] Morimune S, Nishino T, Goto T (2012). Poly(vinyl alcohol)/graphene oxide nanocomposites prepared by a simple eco-process. Polym J.

[59] Gao J, Bao F, Feng L, Shen K, Zhu Q, Wang D, Chen T, Ma R, Yan C (2011). Functionalized graphene oxide modified polysebacic anhydride as drug carrier for levofloxacin controlled release. RSC Adv.

[60] Pan Y, Bao H, Sahoo NG, Wu T, Li L (2011). Water-soluble poly (N‐isopropylacrylamide)–graphene sheets synthesized via click chemistry for drug delivery. Adv Funct Mater.

[61] Zhang L, Lu Z, Zhao Q, Huang J, Shen H, Zhang Z (2011). Enhanced chemotherapy efficacy by sequential delivery of siRNA and anticancer drugs using PEI-grafted graphene oxide. Small.

[62] Chen B, Liu M, Zhang L, Huang J, Yao J, Zhang Z (2011). Polyethylenimine-functionalized graphene oxide as an efficient gene delivery vector. J Mater Chem.

[63] Depan D, Shah J, Misra RDK (2011). Controlled release of drug from folate-decorated and graphene mediated drug delivery system: synthesis, loading efficiency, and drug release response. Mater Sci Engineering: C.

[64] Bao H, Pan Y, Ping Y, Sahoo NG, Wu T, Li L, Li J, Gan LH (2011). Chitosan-functionalized graphene oxide as a nanocarrier for drug and gene delivery. Small.

[65] Hu H, Yu J, Li Y, Zhao J, Dong H (2012). Engineering of a novel pluronic F127/graphene nanohybrid for pH responsive drug delivery. J Biomedical Mater Res Part A.

[66] Li R, Wang Y, Du J, Wang X, Duan A, Gao R, Liu J, Li B (2021). Graphene oxide loaded with tumor-targeted peptide and anti-cancer drugs for cancer target therapy. Sci Rep.

[67] Wei G, Yan M, Dong R, Wang D, Zhou X, Chen J, Hao J (2012). Covalent modification of reduced graphene oxide by means of diazonium chemistry and use as a drug-delivery system, Chemistry (Weinheim an Der Bergstrasse. Germany).

[68] Wang X, Han Q, Yu N, Li J, Yang L, Yang R, Wang C (2015). Aptamer–conjugated graphene oxide–gold nanocomposites for targeted chemo-photothermal therapy of cancer cells. J Mater Chem B.

[69] Wei Y, Zhou F, Zhang D, Chen Q, Xing D (2016). A graphene oxide based smart drug delivery system for tumor mitochondria-targeting photodynamic therapy. Nanoscale.

[70] Guo W, Chen Z, Feng X, Shen G, Huang H, Liang Y, Zhao B, Li G, Hu Y (2021). Graphene oxide (GO)-based nanosheets with combined chemo/photothermal/photodynamic therapy to overcome gastric cancer (GC) paclitaxel resistance by reducing mitochondria-derived adenosine-triphosphate (ATP). J Nanobiotechnol.

[71] Yang Y-Y, Zhang W, Liu H, Jiang J-J, Wang W-J, Jia Z-Y. Cell-penetrating peptide-modified graphene oxide nanoparticles loaded with rictor siRNA for the treatment of triple-negative breast cancer. Drug Design, Development and Therapy; 2021. pp. 4961–72. 10.2147/DDDT.S330059 PMC8671723 34916779

[72] Fan X, Jiao G, Zhao W, Jin P, Li X (2013). Magnetic Fe3O4–graphene composites as targeted drug nanocarriers for pH-activated release. Nanoscale.

[73] Deb A, Andrews NG, Raghavan V (2018). Natural polymer functionalized graphene oxide for co-delivery of anticancer drugs: In-vitro and in-vivo. Int J Biol Macromol.

[74] Deb A, Vimala R (2018). Camptothecin loaded graphene oxide nanoparticle functionalized with polyethylene glycol and folic acid for anticancer drug delivery. J Drug Deliv Sci Technol.

[75] Liu L, Wei Y, Zhai S, Chen Q, Xing D (2015). Dihydroartemisinin and transferrin dual-dressed nano-graphene oxide for a pH-triggered chemotherapy. Biomaterials.

[76] Matiyani M, Rana A, Pal M, Rana S, Melkani AB, Sahoo NG (2022). Polymer grafted magnetic graphene oxide as a potential nanocarrier for pH-responsive delivery of sparingly soluble quercetin against breast cancer cells. RSC Adv.

[77] Hussien N.A., Işıklan N., Türk M. Pectin-conjugated magnetic graphene oxide nanohybrid as a novel drug carrier for paclitaxel delivery. Artif Cells Nanomed Biotechnol. 2018;46:264–73. 10.1080/21691401.2017.1421211 29298530

[78] Rao Z, Ge H, Liu L, Zhu C, Min L, Liu M, Fan L, Li D (2018). Carboxymethyl cellulose modified graphene oxide as pH-sensitive drug delivery system. Int J Biol Macromol.

[79] Parvaneh S, Pourmadadi M, Abdouss M, Pourmousavi SA, Yazdian F, Rahdar A, Díez-Pascual AM (2023). Carboxymethyl cellulose/starch/reduced graphene oxide composite as a pH-sensitive nanocarrier for curcumin drug delivery. Int J Biol Macromol.

[80] Wen H, Dong C, Dong H, Shen A, Xia W, Cai X, Song Y, Li X, Li Y, Shi D (2012). Engineered redox-responsive PEG detachment mechanism in PEGylated nano‐graphene oxide for intracellular drug delivery. Small.

[81] Chen H, Wang Z, Zong S, Wu L, Chen P, Zhu D, Wang C, Xu S, Cui Y (2014). SERS-Fluorescence monitored drug release of a redox-responsive Nanocarrier based on Graphene Oxide in Tumor cells. ACS Appl Mater Interfaces.

[82] Al-Nahain A, Lee SY, In I, Lee KD, Park SY (2013). Triggered pH/redox responsive release of doxorubicin from prepared highly stable graphene with thiol grafted Pluronic. Int J Pharm.

[83] Cho Y, Choi Y (2012). Graphene oxide–photosensitizer conjugate as a redox-responsive theranostic agent. Chem Commun.

[84] Bardajee GR, Hooshyar Z, Farsi M, Mobini A, Sang G (2017). Synthesis of a novel thermo/pH sensitive nanogel based on salep modified graphene oxide for drug release. Mater Sci Engineering: C.

[85] Wang C, Mallela J, Garapati US, Ravi S, Chinnasamy V, Girard Y, Howell M, Mohapatra S (2013). A chitosan-modified graphene nanogel for noninvasive controlled drug release. Nanomed Nanotechnol Biol Med.

[86] Makharza S.A., Cirillo G, Vittorio O, Valli E, Voli F, Farfalla A, Curcio M, Iemma F, Nicoletta F.P., El-Gendy A.A. Magnetic graphene oxide nanocarrier for targeted delivery of cisplatin: a perspective for glioblastoma treatment. Pharmaceuticals. 2019;12:76. 10.3390/ph12020076 PMC6631527 31109098

[87] Pramanik N, Ranganathan S, Rao S, Suneet K, Jain S, Rangarajan A, Jhunjhunwala S (2019). A composite of hyaluronic acid-modified graphene oxide and iron oxide nanoparticles for targeted drug delivery and magnetothermal therapy. ACS Omega.

[88] Ma X, Tao H, Yang K, Feng L, Cheng L, Shi X, Li Y, Guo L, Liu Z (2012). A functionalized graphene oxide-iron oxide nanocomposite for magnetically targeted drug delivery, photothermal therapy, and magnetic resonance imaging. Nano Res.

[89] Swain AK, Pradhan L, Bahadur D (2015). Polymer stabilized Fe3O4-Graphene as an amphiphilic drug carrier for Thermo-Chemotherapy of Cancer. ACS Appl Mater Interfaces.

[90] Guo L, Shi H, Wu H, Zhang Y, Wang X, Wu D, An L, Yang S (2016). Prostate cancer targeted multifunctionalized graphene oxide for magnetic resonance imaging and drug delivery. Carbon.

[91] Wang C, Ravi S, Garapati US, Das M, Howell M, Mallela J, Alwarappan S, Mohapatra SS, Mohapatra S (2013). Multifunctional chitosan magnetic-graphene (CMG) nanoparticles: a theranostic platform for tumor-targeted co-delivery of drugs, genes and MRI contrast agents. J Mater Chem B.

[92] Wang C, Li B, Niu W, Hong S, Saif B, Wang S, Dong C (2015). Shuang, β-Cyclodextrin modified graphene oxide–magnetic nanocomposite for targeted delivery and pH-sensitive release of stereoisomeric anti-cancer drugs. RSC Adv.

[93] Xie M, Zhang F, Peng H, Zhang Y, Li Y, Xu Y, Xie J (2019). Layer-by-layer modification of magnetic graphene oxide by chitosan and sodium alginate with enhanced dispersibility for targeted drug delivery and photothermal therapy. Colloids Surf B.

[94] Shirvalilou S, Khoei S, Khoee S, Raoufi NJ, Karimi MR, Shakeri-Zadeh A (2018). Development of a magnetic nano-graphene oxide carrier for improved glioma-targeted drug delivery and imaging: in vitro and in vivo evaluations. Chemico-Biol Interact.

[95] Gupta J, Prakash A, Jaiswal MK, Agarrwal A, Bahadur D (2018). Superparamagnetic iron oxide-reduced graphene oxide nanohybrid-a vehicle for targeted drug delivery and hyperthermia treatment of cancer. J Magn Magn Mater.

[96] Wang Y, Wang H, Liu D, Song S, Wang X, Zhang H (2013). Graphene oxide covalently grafted upconversion nanoparticles for combined NIR mediated imaging and photothermal/photodynamic cancer therapy. Biomaterials.

[97] Tran TH, Nguyen HT, Pham TT, Choi JY, Choi HG, Yong CS, Kim JO (2015). Development of a Graphene Oxide Nanocarrier for Dual-Drug Chemo-Phototherapy to overcome Drug Resistance in Cancer. ACS Appl Mater Interfaces.

[98] Shi J, Wang B, Chen Z, Liu W, Pan J, Hou L, Zhang Z (2016). A multi-functional Tumor Theranostic Nanoplatform for MRI guided Photothermal-Chemotherapy. Pharm Res.

[99] Song J, Yang X, Jacobson O, Lin L, Huang P, Niu G, Ma Q, Chen X (2015). Sequential drug release and enhanced Photothermal and Photoacoustic Effect of Hybrid reduced Graphene Oxide-Loaded Ultrasmall Gold Nanorod vesicles for Cancer Therapy. ACS Nano.

[100] Vinothini K, Rajendran NK, Rajan M, Ramu A, Marraiki N, Elgorban AM (2020). A magnetic nanoparticle functionalized reduced graphene oxide-based drug carrier system for a chemo-photodynamic cancer therapy. New J Chem.

[101] Lu SL, Wang YH, Liu GF, Wang L, Li Y, Guo ZY, Cheng C (2021). Graphene Oxide nanoparticle-loaded Ginsenoside Rg3 improves photodynamic therapy in inhibiting malignant progression and stemness of Osteosarcoma. Front Mol Biosci.

[102] Karimi M, Eslami M, Sahandi-Zangabad P, Mirab F, Farajisafiloo N, Shafaei Z, Ghosh D, Bozorgomid M, Dashkhaneh F, Hamblin MR (2016). pH‐Sensitive stimulus‐responsive nanocarriers for targeted delivery of therapeutic agents. Wiley Interdisciplinary Reviews: Nanomed Nanobiotechnol.

[103] Kavitha T, Abdi SIH, Park S-Y (2013). pH-sensitive nanocargo based on smart polymer functionalized graphene oxide for site-specific drug delivery. Phys Chem Chem Phys.

[104] Sun X, Liu Z, Welsher K, Robinson JT, Goodwin A, Zaric S, Dai H (2008). Nano-graphene oxide for cellular imaging and drug delivery. Nano Res.

[105] Guo Y, Xu H, Li Y, Wu F, Li Y, Bao Y, Yan X, Huang Z, Xu P (2017). Hyaluronic acid and arg-gly-asp peptide modified Graphene oxide with dual receptor-targeting function for cancer therapy. J Biomater Appl.

[106] Wang C, Chen B, Zou M, Cheng G (2014). Cyclic RGD-modified chitosan/graphene oxide polymers for drug delivery and cellular imaging. Colloids Surf B.

[107] Boddu A, Reddy OS, Zhang D, Rao KK, Lai W-F (2022). ROS-generating, pH-responsive and highly tunable reduced graphene oxide-embedded microbeads showing intrinsic anticancer properties and multi-drug co-delivery capacity for combination cancer therapy. Drug Delivery.

[108] Cheng R, Feng F, Meng F, Deng C, Feijen J, Zhong Z (2011). Glutathione-responsive nano-vehicles as a promising platform for targeted intracellular drug and gene delivery. J Controlled Release.

[109] Zhao X, Yang L, Li X, Jia X, Liu L, Zeng J, Guo J, Liu P (2015). Functionalized Graphene Oxide nanoparticles for Cancer Cell-Specific Delivery of Antitumor Drug. Bioconjug Chem.

[110] Ma N, Song A, Li Z, Luan Y (2019). Redox-Sensitive Prodrug molecules Meet Graphene Oxide: an efficient graphene oxide-based nanovehicle toward Cancer Therapy. ACS Biomaterials Sci Eng.

[111] Kim DJ, Kim J, Lee HL, Lee S, Choi JS, Kim SJ, Jeong YI, Kang DH (2018). Redox-Responsive nanocomposites composed of Graphene Oxide and Chlorin e6 for photodynamic treatment of Cholangiocarcinoma. Bull Korean Chem Soc.

[112] Yin T, Liu J, Zhao Z, Zhao Y, Dong L, Yang M, Zhou J, Huo M (2017). Redox sensitive hyaluronic acid-decorated graphene oxide for photothermally controlled tumor‐cytoplasm‐selective rapid drug delivery. Adv Funct Mater.

[113] Seo HI, Cheon YA, Chung BG (2016). Graphene and Thermo-responsive polymeric nanocomposites for therapeutic applications. Biomed Eng Lett.

[114] Farjadian F, Ghasemi S, Andami Z, Tamami B (2020). Thermo-responsive nanocarrier based on poly (N-isopropylacrylamide) serving as a smart doxorubicin delivery system. Iran Polym J.

[115] Khanizadeh L, Sarvari R, Massoumi B, Agbolaghi S, Beygi-Khosrowshahi Y (2020). Dual nano-carriers using polylactide-block-poly (n-isopropylacrylamide-random-acrylic acid) polymerized from reduced graphene oxide surface for doxorubicin delivery applications. J Ultrafine Grained Nanostructured Mater.

[116] Khoee S, Karimi MR (2018). Dual-drug loaded Janus graphene oxide-based thermoresponsive nanoparticles for targeted therapy. Polymer.

[117] Liang J, Chen B, Hu J, Huang Q, Zhang D, Wan J, Hu Z, Wang B (2019). pH and Thermal Dual-Responsive Graphene Oxide nanocomplexes for targeted drug delivery and Photothermal-Chemo/Photodynamic synergetic therapy. ACS Appl Bio Mater.

[118] Huang D, Sun L, Huang L, Chen Y (2021). Nanodrug delivery systems modulate tumor vessels to increase the enhanced permeability and retention effect. J Personalized Med.

[119] Zhang B, Yu Q, Liu Y (2020). Alternating magnetic field controlled targeted drug delivery based on Graphene Oxide-Grafted Nanosupramolecules. Chemistry–A Eur J.

[120] Yang X, Wang Y, Huang X, Ma Y, Huang Y, Yang R, Duan H, Chen Y (2011). Multi-functionalized graphene oxide based anticancer drug-carrier with dual-targeting function and pH-sensitivity. J Mater Chem.

[121] Wang Z, Zhou C, Xia J, Via B, Xia Y, Zhang F, Li Y, Xia L (2013). Fabrication and characterization of a triple functionalization of graphene oxide with Fe3O4, folic acid and doxorubicin as dual-targeted drug nanocarrier. Colloids Surf B.

[122] Işıklan N, Hussien NA, Türk M (2021). Synthesis and drug delivery performance of gelatin-decorated magnetic graphene oxide nanoplatform. Colloids Surf a.

[123] Li D, Deng M, Yu Z, Liu W, Zhou G, Li W, Wang X, Yang D-P, Zhang W (2018). Biocompatible and stable GO-Coated Fe3O4 nanocomposite: a robust drug delivery carrier for Simultaneous Tumor MR Imaging and targeted therapy. ACS Biomaterials Sci Eng.

[124] Chawda N, Basu M, Majumdar D, Poddar R, Mahapatra SK, Banerjee I (2019). Engineering of Gadolinium-decorated Graphene Oxide nanosheets for Multimodal Bioimaging and Drug Delivery. ACS Omega.

[125] Gonzalez-Rodriguez R, Campbell E, Naumov A (2019). Multifunctional graphene oxide/iron oxide nanoparticles for magnetic targeted drug delivery dual magnetic resonance/fluorescence imaging and cancer sensing. PLoS ONE.

[126] Song J, Qu J, Swihart MT, Prasad PN (2016). Near-IR responsive nanostructures for nanobiophotonics: emerging impacts on nanomedicine. Nanomed Nanotechnol Biol Med.

[127] Zhou L, Jiang H, Wei S, Ge X, Zhou J, Shen J (2012). High-efficiency loading of hypocrellin B on graphene oxide for photodynamic therapy. Carbon.

[128] Tian B, Wang C, Zhang S, Feng L, Liu Z (2011). Photothermally enhanced photodynamic therapy delivered by nano-graphene oxide. ACS Nano.

[129] Kim H, Lee D, Kim J, Kim T-i, Kim WJ (2013). Photothermally triggered cytosolic drug delivery via endosome disruption using a Functionalized reduced Graphene Oxide. ACS Nano.

[130] Kurapati R, Raichur AM (2013). Near-infrared light-responsive graphene oxide composite multilayer capsules: a novel route for remote controlled drug delivery. Chem Commun.

[131] Du P, Yan J, Long S, Xiong H, Wen N, Cai S, Wang Y, Peng D, Liu Z, Liu Y (2020). Tumor microenvironment and NIR laser dual-responsive release of berberine 9-O-pyrazole alkyl derivative loaded in graphene oxide nanosheets for chemo-photothermal synergetic cancer therapy. J Mater Chem B.

[132] Liu W, Zhang X, Zhou L, Shang L, Su Z (2019). Reduced graphene oxide (rGO) hybridized hydrogel as a near-infrared (NIR)/pH dual-responsive platform for combined chemo-photothermal therapy. J Colloid Interface Sci.

[133] Ha W, Zhao X-B, Jiang K, Kang Y, Chen J, Li B-J, Shi Y-P (2016). A three-dimensional graphene oxide supramolecular hydrogel for infrared light-responsive cascade release of two anticancer drugs. Chem Commun.

[134] Wang P, Huang C, Xing Y, Fang W, Ren J, Yu H, Wang G. NIR-light-and pH-responsive graphene oxide hybrid cyclodextrin-based supramolecular hydrogels, Langmuir, 35 (2019) 1021–1031. 10.1021/acs.langmuir.8b03689 30621394

[135] Wu J, Li Z, Li Y, Pettitt A, Zhou F (2018). Photothermal effects of reduced Graphene Oxide on Pancreatic Cancer. Technol Cancer Res Treat.

[136] Cheon Y.A., Bae J.H., Chung B.G. Reduced Graphene Oxide Nanosheet for Chemo-Photothermal Therapy. Langmuir. 2016;32:2731–6. 10.1021/acs.langmuir.6b00315 26930106

[137] Lim JH, Kim DE, Kim E-J, Ahrberg CD, Chung BG (2018). Functional graphene oxide-based nanosheets for Photothermal Therapy. Macromol Res.

[138] Dolatkhah M, Hashemzadeh N, Barar J, Adibkia K, Aghanejad A, Barzegar-Jalali M, Omidian H, Omidi Y (2021). Stimuli-responsive graphene oxide and methotrexate-loaded magnetic nanoparticles for breast cancer-targeted therapy. Nanomedicine.

[139] Park JH, Yoon J-K, Kim Y-J, Lee T-J, Jeong G-J, Kim D-I, Bhang SH (2019). Enhancing therapeutic efficacy of photothermal therapy using poloxamer-reduced graphene oxide and mesenchymal stem cells. J Ind Eng Chem.

[140] Shao L, Zhang R, Lu J, Zhao C, Deng X, Wu Y (2017). Mesoporous silica coated polydopamine functionalized reduced Graphene Oxide for Synergistic targeted chemo-photothermal therapy. ACS Appl Mater Interfaces.

[141] Jiang W, Mo F, Lin Y, Wang X, Xu L, Fu F (2018). Tumor targeting dual stimuli responsive controllable release nanoplatform based on DNA-conjugated reduced graphene oxide for chemo-photothermal synergetic cancer therapy. J Mater Chem B.

[142] Xu X, Wang J, Wang Y, Zhao L, Li Y, Liu C (2018). Formation of graphene oxide-hybridized nanogels for combinative anticancer therapy.

[143] Tang Y, Hu H, Zhang MG, Song J, Nie L, Wang S, Niu G, Huang P, Lu G, Chen X (2015). An aptamer-targeting photoresponsive drug delivery system using off–on graphene oxide wrapped mesoporous silica nanoparticles. Nanoscale.

[144] Fan H-y, Yu X-h, Wang K, Yin Y-j, Tang Y-j, Tang Y-l (2019). -h. Liang, Graphene quantum dots (GQDs)-based nanomaterials for improving photodynamic therapy in cancer treatment. Eur J Med Chem.

[145] Qidwai A, Nabi B, Kotta S, Narang JK, Baboota S, Ali J (2020). Role of nanocarriers in photodynamic therapy. Photodiagn Photodyn Ther.

[146] Sahu A, Choi WI, Lee JH, Tae G (2013). Graphene oxide mediated delivery of methylene blue for combined photodynamic and photothermal therapy. Biomaterials.

[147] Gulzar A, Xu J, Yang D, Xu L, He F, Gai S, Yang P (2018). Nano-graphene oxide-UCNP-Ce6 covalently constructed nanocomposites for NIR-mediated bioimaging and PTT/PDT combinatorial therapy. Dalton Trans.

[148] Huang P, Xu C, Lin J, Wang C, Wang X, Zhang C, Zhou X, Guo S, Cui D (2011). Folic acid-conjugated graphene oxide loaded with photosensitizers for targeting photodynamic therapy. Theranostics.

[149] Zhou L, Wei S, Ge X, Zhou J, Jiang H, Li F, Shen J (2014). Combination of chemotherapy and photodynamic therapy using graphene oxide as drug delivery system. J Photochem Photobiol B.

[150] Huang X, Chen J, Wu W, Yang W, Zhong B, Qing X, Shao Z (2020). Delivery of MutT homolog 1 inhibitor by functionalized graphene oxide nanoparticles for enhanced chemo-photodynamic therapy triggers cell death in osteosarcoma. Acta Biomater.

[151] Ding Y-F, Kwong CHT, Li S, Pan Y-T, Wei J, Wang L-H, Mok GSP, Wang R (2021). Supramolecular nanomedicine derived from cucurbit[7]uril-conjugated nano-graphene oxide for multi-modality cancer therapy. Biomaterials Sci.

[152] Duan G, Zhang Y, Luan B, Weber JK, Zhou RW, Yang Z, Zhao L, Xu J, Luo J, Zhou R (2017). Graphene-Induced Pore Formation Cell Membr Sci Rep.

[153] Liao C, Li Y, Tjong SC. Graphene nanomaterials: synthesis, Biocompatibility, and cytotoxicity. Int J Mol Sci, 19 (2018). 10.3390/ijms19113564 PMC6274822 30424535

[154] Bach D, Wachtel E (2003). Phospholipid/cholesterol model membranes: formation of cholesterol crystallites. Biochim et Biophys Acta (BBA) - Biomembr.

[155] Liu JH, Yang ST, Wang H, Chang Y, Cao A, Liu Y (2012). Effect of size and dose on the biodistribution of graphene oxide in mice. Nanomed (London England).

[156] Mohamed HRH, Welson M, Yaseen AE, El-Ghor AA (2020). Estimation of genomic instability and mutation induction by graphene oxide nanoparticles in mice liver and brain tissues. Environ Sci Pollut Res Int.

[157] Patlolla AK, Randolph J, Kumari SA, Tchounwou PB (2016). Toxicity evaluation of Graphene Oxide in kidneys of Sprague-Dawley rats. Int J Environ Res Public Health.

[158] Wang A, Pu K, Dong B, Liu Y, Zhang L, Zhang Z, Duan W, Zhu Y (2013). Role of surface charge and oxidative stress in cytotoxicity and genotoxicity of graphene oxide towards human lung fibroblast cells. J Appl Toxicology: JAT.

[159] Pelin M, Fusco L, Martín C, Sosa S, Frontiñán-Rubio J, González-Domínguez JM, Durán-Prado M, Vázquez E, Prato M, Tubaro A (2018). Graphene and graphene oxide induce ROS production in human HaCaT skin keratinocytes: the role of xanthine oxidase and NADH dehydrogenase. Nanoscale.

